# Rewriting the Central European Early Bronze Age Chronology: Evidence from Large-Scale Radiocarbon Dating

**DOI:** 10.1371/journal.pone.0139705

**Published:** 2015-10-21

**Authors:** Philipp W. Stockhammer, Ken Massy, Corina Knipper, Ronny Friedrich, Bernd Kromer, Susanne Lindauer, Jelena Radosavljević, Fabian Wittenborn, Johannes Krause

**Affiliations:** 1 Cluster of Excellence “Asia and Europe in a Global Context”, Heidelberg University, Heidelberg, Germany; 2 Institute for Prehistory and Early History and Near Eastern Archaeology, Heidelberg University, Heidelberg, Germany; 3 Ludwig-Maximilians-University, Munich, Germany; 4 Heidelberg Academy of Sciences, Heidelberg, Germany; 5 Curt Engelhorn Center for Archaeometry gGmbH, Mannheim, Germany; 6 Max Planck Institute for the Science of Human History, Jena, Germany; University at Buffalo, UNITED STATES

## Abstract

The transition from the Neolithic to the Early Bronze Age in Central Europe has often been considered as a supra-regional uniform process, which led to the growing mastery of the new bronze technology. Since the 1920s, archaeologists have divided the Early Bronze Age into two chronological phases (Bronze A1 and A2), which were also seen as stages of technical progress. On the basis of the early radiocarbon dates from the cemetery of Singen, southern Germany, the beginning of the Early Bronze Age in Central Europe was originally dated around 2300/2200 BC and the transition to more complex casting techniques (i.e., Bronze A2) around 2000 BC. On the basis of 140 newly radiocarbon dated human remains from Final Neolithic, Early and Middle Bronze Age cemeteries south of Augsburg (Bavaria) and a re-dating of ten graves from the cemetery of Singen, we propose a significantly different dating range, which forces us to re-think the traditional relative and absolute chronologies as well as the narrative of technical development. We are now able to date the beginning of the Early Bronze Age to around 2150 BC and its end to around 1700 BC. Moreover, there is no transition between Bronze (Bz) A1 and Bronze (Bz) A2, but a complete overlap between the type objects of the two phases from 1900–1700 BC. We thus present a revised chronology of the assumed diagnostic type objects of the Early Bronze Age and recommend a radiocarbon-based view on the development of the material culture. Finally, we propose that the traditional phases Bz A1 and Bz A2 do not represent a chronological sequence, but regionally different social phenomena connected to the willingness of local actors to appropriate the new bronze technology.

## Introduction

The impact of technical innovations on the development of societies has been of central interest since the beginning of archaeological research. V. Gordon Childe (1925) [1] already considered technical innovation as a crucial factor for societal changes in prehistory. However, the notion that past societies immediately accepted innovations is highly problematic and deeply rooted in modern notions of progress. The transition from the Late Neolithic (LN) to the Early Bronze Age (EBA) in Central Europe has long been considered as a linear evolutionary development that led to a growing mastery of the new technology. Paul Reinecke [2] was the first to define the Central European EBA as Bz A and some years later further divided this phase into two chronological sub-phases–namely Bz A1 and Bz A2 –which were also seen as states of technical progress [3]. All subsequent chronological discussion has been based on Reinecke’s work and many scholars have also kept the idea of a gradual development of the technology from hammered metal objects–consisting mostly of copper with hardly any tin in Bz A1 –up to perfectly alloyed bronze with 90% of copper and 10% of tin and refined casting techniques in Bz A2. Critics have argued for a more complex, non-linear history of the spread and appropriation of bronze technology [4] [5] [6] [7]. They have emphasized the relevance of social factors in this process and pointed to cases of belated appropriation of metallurgy. Moreover, scholars have already pointed to the fact that differences within Únětice material culture could be the result of social or spatial phenomena rather than chronological ones [8] [9] [10]. Others have doubted the applicability of the Reinecke system for the area of the Únětice culture [11]. Nevertheless, the small number of existing ^14^C dates have not so far substantiated these important doubts. Despite these critical voices, researchers have continuously tried to synchronize the Reinecke system with the chronologies of the Únětice culture [12] [13]. With regard to Early Bronze Age material culture from Central Europe, a similar evolutionist perspective still prevails: simple bone objects–especially pins–were proposed to be the starting point of this development as their shapes seemed to foreshadow the later metal shapes [14]. Walter Ruckdeschel presented a more detailed view in 1978 [15]. He proposed a further subdivision of Bz A1 and A2 on the basis of particular types of pins, which still is the basis of today’s relative chronology for southern Germany. However, as no grave contained more than one pin, the supposed development of the pins could not be crosschecked with the help of associations of different types of pins. Since the 1990s, Stephan Möslein [16] and Wolfgang David [17] have tried to further develop and refine Ruckdeschel’s chronology by taking hoard finds and pottery from settlements into account, but did not question the general chronological sequence of the pins as suggested by Ruckdeschel. Since then, it has already been acknowledged that only a large series of ^14^C-dated contexts may enable us to solve these long-standing problems of understanding cultural developments in the EBA.

The linking of the relative chronological system with absolute dates is of major importance to understand the temporal dimension of the respective phases. Until the 1980s, the beginning of the EBA in Central Europe was dated to around 1700 BC on the basis of long-distance connections with the Mediterranean (for an overview cf. [18]). Since the late 1980s, a growing number of radiocarbon-dated contexts have enabled us to obtain scientifically determined dates of various phases. When Rüdiger Krause [19] published the radiocarbon dates for the Bz A1 cemetery of Singen in southern Germany, archaeologists were electrified by the surprisingly old dates for the beginning of the EBA. Following Becker et al. [20] it soon became common sense to locate the beginning of the EBA around 2300 BC or 2200 BC in central Europe at the very latest [21], [22]. As these dates enabled archaeologists to replace the insecurities of long-distance dating by the seemingly scientific truth of ^14^C dates, the new early dates were accepted widely and found their way into museum exhibitions as well as popular literature. At the same time in the late 1980s, dendrochronological dating of the so-called princely grave of Leubingen in Saxony-Anhalt helped to identify the beginning of Bz A2 in the 20^th^ century BC and dendro-dates from Alpine lakeside settlements suggested an end of the EBA in the 16^th^ century BC [23], [24]. Therefore, it seemed natural to date the transition from Bz A1 to Bz A2 to around 2000 BC.

Since then, new radiocarbon dates have produced more problems rather than improving our understanding of the connection between the relative and absolute chronology: 22 graves from the area around Stuttgart in southern Germany, which were typologically attributed to Bz A1, were dated to the 20^th^ and 19^th^ centuries BC and, therefore, seemingly too young [20], [25]. On the other hand, contexts with Bz A2 type objects in the area of the Únětice culture (*Aunjetitzer Kultur*) in eastern Germany were dated to the centuries before 2000 BC ([26], [10], [27]): e.g. Quenstedt grave 34 with an *Ösenkopfnadel* (eyelet pin): 2350–1907 BC (90.7% probability); hoard II of Melz: 2205–1952 BC (95.4% probability). These results also met criticism, as it seemed impossible to have such early dates for sophistically cast bronze objects [28], though the early date for a grave with Bz A2 bronzes from Feuersbrunn in Austria– 2198–2162 BC (8.4% probability) and 2152–1960 BC (87.0% probability)–further underlines the early appearance of Bz A2 types [29]. However, these contradicting results have not found adequate explanation so far. Moreover, the rarity of Bz A1 types in eastern Germany and the rare Bz A2 types in southern Germany should have already raised the question, whether the traditional EBA relative chronology can be applied for the whole of Central Europe without further modification [30]. Until now, the lack of radiocarbon dated Bz A1 and A2 type objects from different contexts from southern respectively eastern Germany has prevented us from better understanding EBA chronology as well as social and cultural developments.

## Materials and Methods

**Fig 1:**
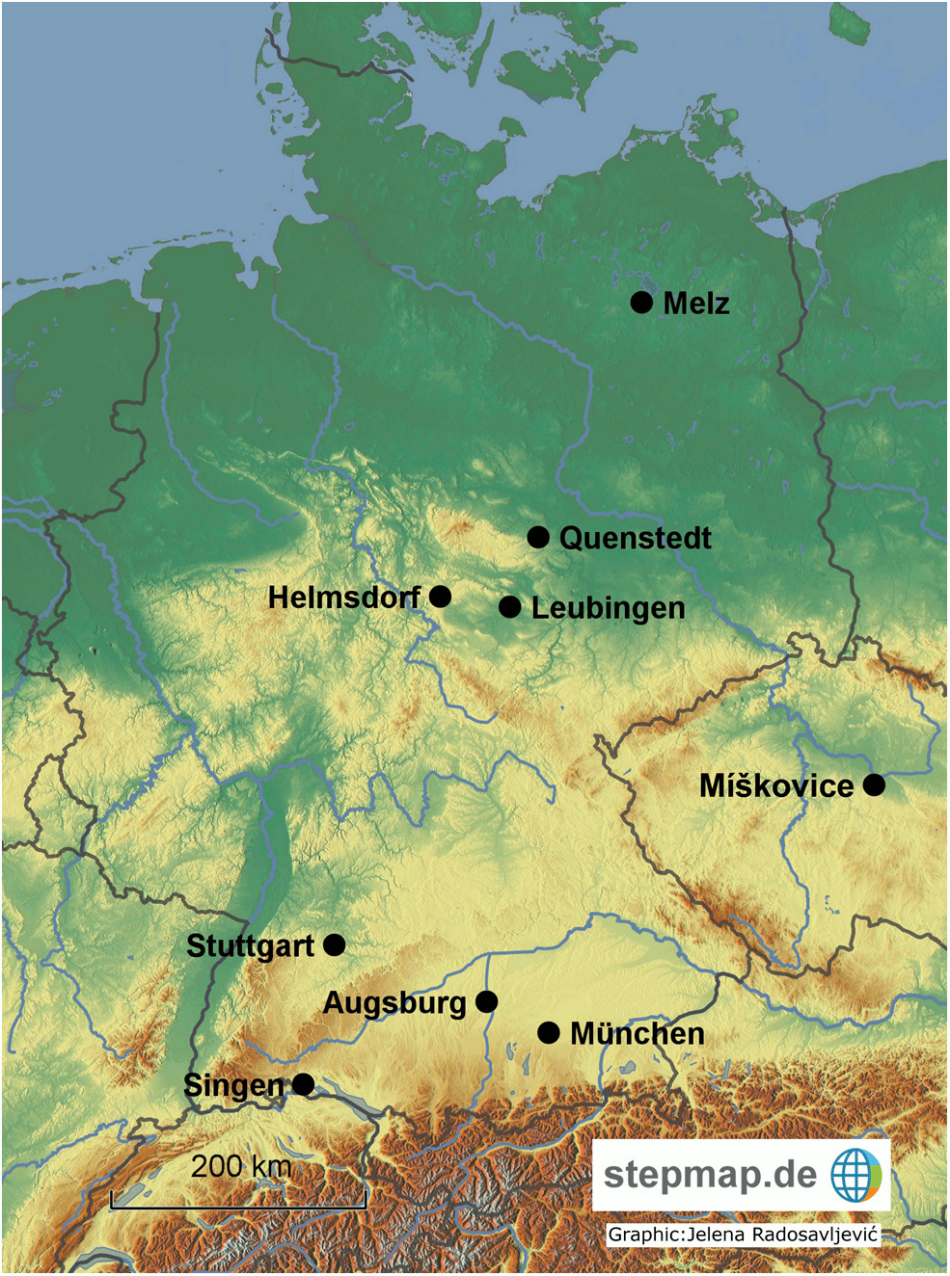
Map of important sites in Germany and Bohemia mentioned in the text.

**Fig 2:**
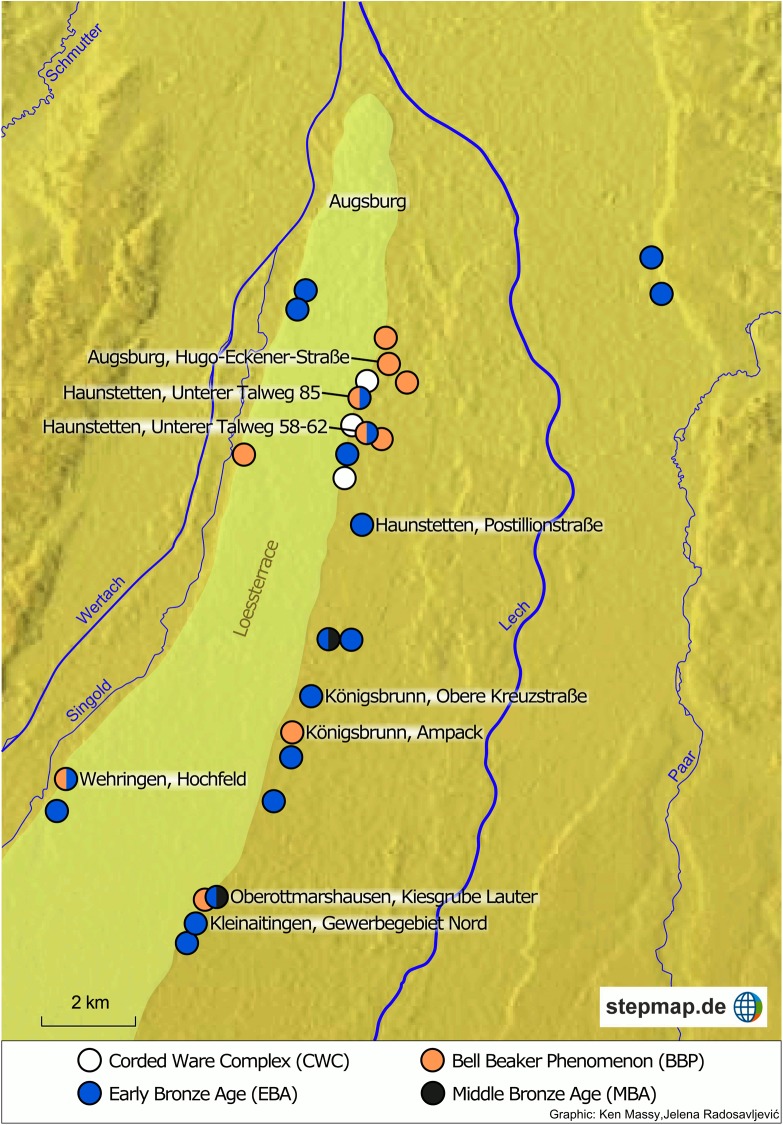
Map of cemeteries and single burial sites south of the city of Augsburg. The labelled sites provide samples included in this study.

Since the 1980s a large number of Late Neolithic (LN) and Early Bronze Age (EBA) graves were excavated south of the present-day city of Augsburg (Bavaria, Germany) (Fig 1), (Fig 2). The LN and EBA cemeteries are arranged like pearls on a necklace and located just outside the eastern and western fringe of a large and extremely fertile loess terrace in the middle of the Lech valley. As a consequence, the cemeteries are placed in a very similar topographic position. On the eastern side of the loess terrace, several cemeteries are associated with a small hamlet placed to the west. Therefore, at least theoretically, all settlements had access to the same natural resources. On the western side of the loess terrace, only cemeteries are known whereas the related settlements have not yet been located. The 32 burial places vary largely in size–from single burials and small cemeteries (such as three EBA graves in Augsburg-Haunstetten, Unterer Talweg 85) up to 63 of them in Kleinaitingen (Table 1). They are either restricted to one chronological phase–LN or EBA up to the beginning Middle Bronze Age (MBA)–or span a longer period of time with Late Neolithic Bell Beaker Phenomenon (BBP) and EBA burials close by each other. Consisting of 390 burials, it presents one of the largest concentrations of LN/EBA/early MBA burials in central Europe. Furthermore, the graves are remarkable for the good preservation of collagen in the bone material as well as rich grave goods–especially metal objects. In contrast to other EBA cemeteries, neither large-scale grave robbery nor insufficiently documented excavations reduce the significance of the evidence.

**Table 1:** Cemeteries from the Augsburg region, the total number of graves and of sampled graves, respectively.

Site	Total number of graves	Sampled graves
**Corded Ware Complex**		
Haunstetten, Siemens-Gelände	2	0
Haunstetten, Unterer Talweg 89	1	0
Haunstetten, Unterer Talweg 121	1	0
**Bell Beaker Phenomenon**		
Augsburg, Bürgermeister-Ulrich-Straße	12	0
Augsburg, Hugo-Eckener-Straße	11	10
Augsburg, Universitätsgelände	24	0
Haunstetten, Im Tal 6	6	0
Haunstetten, Unterer Talweg 58–62	2	2
Haunstetten, Unterer Talweg 85 I (Northern group)	3 or 5	3
Haunstetten, Unterer Talweg 85 II (Southern group)	2	2
Inningen, Libellenweg	2	0
Königsbrunn, Ampack	5	1
Oberottmarshausen, Kiesgrube Lauter	1	0
Wehringen, Hochfeld	1	1
**Early Bronze Age**		
Friedberg, Kissinger Weg (Metzgerwäldchen)	1	0
Friedberg, Rathaus	1	0
Göggingen, Gerhard-Hauptmannstraße	1	0
Göggingen, Richard-Wagner-Straße	1	0
Haunstetten, Postillionstraße	41	22
Haunstetten, Unterer Talweg 58–62	10	7
Haunstetten, Unterer Talweg 85	3	3
Haunstetten, Unterer Talweg 111	10	0
Kleinaitingen, Gewerbegebiet Nord	63	32
Kleinaitingen, Herbst- und Friedensstraße	29	0
Königsbrunn, Kiesgrube Burkhart	13	0
Königsbrunn, Obere Kreuzstraße	48	23
Königsbrunn, Simpertstraße	2	0
Königsbrunn, Steinkistengrab "Oberes Feld"	1	0
Wehringen, Hochfeld	14	9
Wehringen, Mittelunterfeld	1	0
**Early and Middle Bronze Age**		
Königsbrunn, Afra- und Augustusstraße	44	0
Oberottmarshausen, Kiesgrube Lauter	32	21

We selected 140 individuals from 132 LN and EBA burials (eight of them double burials) for radiocarbon dating. The skeletal material from the cemeteries of Haunstetten (Unterer Talweg 58–62; Unterer Talweg 85; Postillionstraße) and Augsburg (Hugo-Eckener-Straße) is stored at the Stadtarchäologie Augsburg; that from Königsbrunn (Obere Kreuzstraße; Ampack), Kleinaitingen and Oberottmarshausen is kept in the Archäologisches Museum Königsbrunn, and the material from Wehringen was situated at the Bayerisches Landesamt für Denkmalpflege, Dienststelle Thierhaupten, at the time of our sampling. All necessary permits were obtained for the described study, which complied with all relevant regulations (Augsburg: permit from 23^rd^ April 2014 by Michaela Hermann; Königsbrunn: permit from 23^rd^ April 2014 by Rainer Linke; Thierhaupten: permit from 18^th^ July 2013 by Hanns Dietrich). The material from Augsburg and Königsbrunn is accessible upon request in the respective collections; that from Wehringen can meanwhile be found in the Anthropologische Staatssammlung, Munich.

Based on archaeological criteria, 19 of them were attributed to the BBP, 102 to the EBA and 19 to the EBA/early MBA in order to establish their chronology. The selection of samples was guided by several considerations: in the case of small cemeteries (e.g. Haunstetten, Unterer Talweg 85), we sampled all individuals with sufficient bone preservation. In the case of larger cemeteries (e.g. Kleinaitingen, Gewerbegebiet Nord), we selected individuals due to the following criteria: burials with chronologically significant or remarkable grave goods (especially pins, daggers and other weapons, *Ösenhalsringe*, elaborated head adornments etc.) as well as a representative number of samples from definable groups of graves and children as well as adults–irrespective of the presence of grave goods. Unfortunately, there is a significant difference of graves with objects datable to Bz A1 and Bz A2, respectively, as the number of graves with Bz A2 type objects is small in the Augsburg region–similar to the rest of southern Germany. As the 2σ calibrated ranges of burials in the Augsburg region cover the complete period of time defined as Early Bronze Age, this lack of Bz A2 type objects must be explained in their infrequent selection as grave goods.

Our first results from Augsburg contradicted some of the older radiocarbon dates from Singen analyzed by radiometric measurement with proportional gas counters (decay counting of ^14^C) in the 1980s [19], especially as the grave goods seemed to be almost identical (e.g. large *Rudernadeln*, *Horkheimer Nadeln*–the pin types are further discussed below). Therefore, we decided to re-evaluate the burials from Singen, which played a crucial role in the understanding of the chronological and cultural development of the EBA [19]. We re-sampled the same individuals, which had already been analyzed in the 1980s [31], and were now able to apply AMS dating. The skeletal material from Singen is currently stored and accessible in the Hegau-Museum in Singen and our sampling was permitted by Joachim Wahl and Jürgen Hald (permit from 10^th^ July 2014). Only the skeletal remains of grave 82 from Singen could not be localized in the archive and could not be dated again. Grave 69 was sampled in addition to the old series (cf. [20], 431 Fig 1 erroneously attributes Hd-8974-9155 to grave 69, although it belongs to grave 65; [19], 171 Tab. 5 is correct).

The old radiometric dates from Singen were obtained in the radiocarbon laboratory of the Heidelberg Academy of Sciences (samples using lab codes Hd), and the new series using AMS was measured in the Klaus-Tschira-Archaeometry-Center, Mannheim, Germany (samples using lab codes MAMS).

The pretreatment procedure for radiometric analysis of the samples included decalcification in weak HCl over several days up to one week and dialysis to remove proteins below 10 kD. The resulting sample material was combusted to CO_2_. The gas was purified and the radiocarbon age was determined radiometrically by decay counting for up to one week in gas proportional counters [32].

The new bone samples taken for AMS dating were decalcified. Bone collagen was obtained in a modified Longin extraction [33], followed by ultrafiltration (30 kD) and freeze-drying. Collagen was combusted in an Elemental Analyzer (Elementar Microcube). CO_2_ was collected cryogenically and converted to graphite using Fe as catalyst. The samples were measured using the MICADAS AMS system of the Mannheim facility [34]. In the Elemental Analyzer the C:N ratio can be determined. For all samples the C:N ratio was in the accepted range of well-preserved bone (2.9–3.6) [35]. The data were calibrated using Oxcal v4.2.24 [36] and IntCal13 [37].

We applied Bayesian modeling (for the method cf. [38] and [36]) in order to calculate the degree of overlap between the different phases on a statistical basis. By combining archaeological information (e.g. the attribution to archaeologically defined entities) with a series of chronometric dates, Bayesian modeling can delineate transitions between phases better than the individual ^14^C dates. In order to study the transition between BBP and EBA, we built a multi-phase model allowing for overlap between these two phases (using Oxcal v.4.2.24 [34]).

Extensive consumption of aquatic food, such as fish or mollusks, may have contributed ^14^C-depleted carbon to the human diet resulting in apparently too old radiocarbon dates. This so-called reservoir effect is well-known for marine diets [33]. It is also a possible concern at inland localities, where carbonates of geological origin contribute carbon with a significantly lower ^14^C concentration to freshwater habitats [39], [40]. The analysis of stable carbon and nitrogen isotope ratios (δ^13^C and δ^15^N) of bone collagen is a well-established method for human diet reconstruction and may indicate significant contribution of aquatic food items [41]. In order to identify individuals whose dietary habits may have influenced the results of radiocarbon dating we determined the stable isotope ratios of bone collagen of 43 individuals from the Augsburg region. They include all individuals of the BBP with sufficient collagen preservation (n = 18) and a selection of 25 EBA individuals with diagnostic pin types. The light stable isotope analyses for Singen were conducted at the Max Planck Institute for Evolutionary Anthropology in Leipzig and published in summary in [42]. Four individual values were adapted from [43].

The stable isotope analyses for the individuals from the Augsburg region were carried out on collagen extracts remaining after radiocarbon dating. C and N contents were determined by elemental analyzer (vario EL III, Elementar Analytical Systems) and isotope compositions by an IsoPrime High Performance IRMS (VG Instruments) at the Institute for Organic Chemistry at the University of Mainz, Germany. All measurements were performed in duplicate, and the results reported in δ-notation in ‰ relative to VPDB for carbon and to AIR for nitrogen. The raw data were normalized using two-point calibrations based on USGS 40 and IAEA N2 for nitrogen and IAEA CH6 and CH7 for carbon [44]. Measurement errors are less than ± 0.2 ‰ for nitrogen and ± 0.1 ‰ for carbon.

## Results

In the discussion that follows, we present the results of the radiocarbon dating in chronological order for the Augsburg region. We bring them together with the traditional archaeological division of the respective phases in order to connect traditional relative-chronological phases with the absolute-chronological evidence. These results are then compared with the old and new dates from Singen.

### Augsburg Region

**Fig 3:**
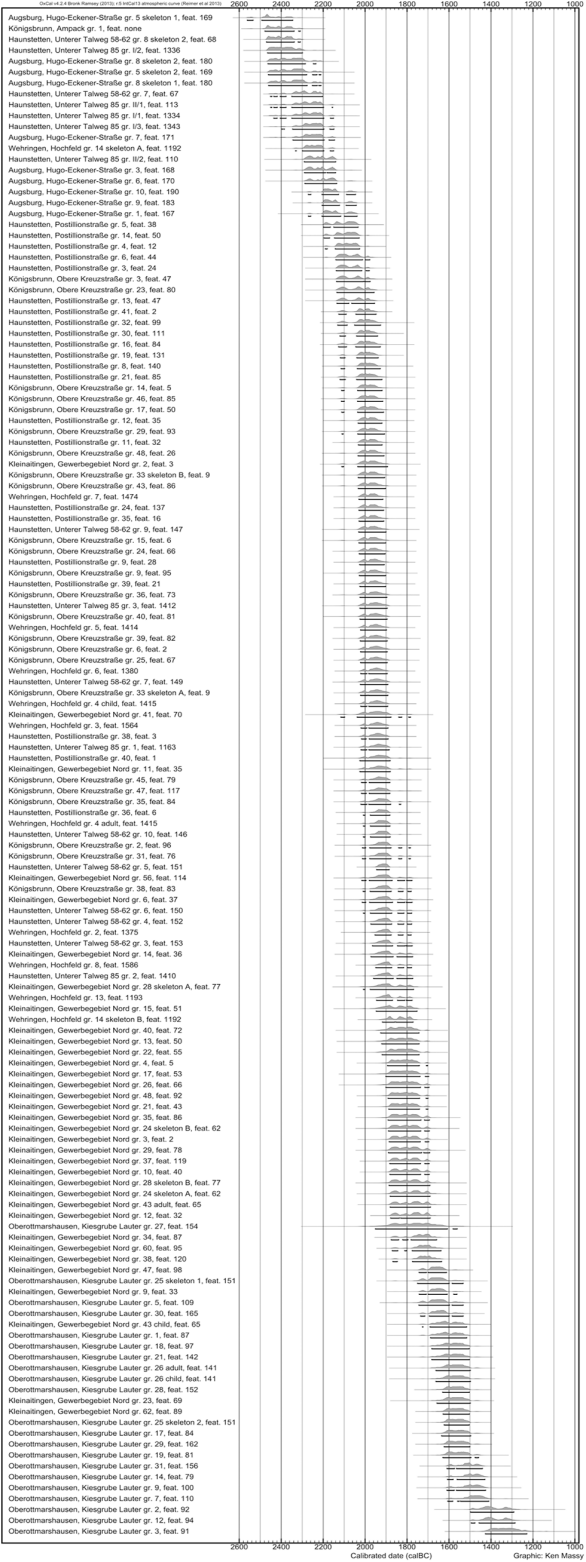
Plot of all radiocarbon dates from burials of the Augsburg region. The brackets mark the 2σ calibrated ranges.

The earliest dates for graves of the BBP in the Augsburg region start with their 2σ calibrated range already in the 25^th^ century BC–e.g. Königsbrunn, Ampack gr. 1: 2478–2339 BC (94.4% probability) and 2317–2310 BC (1.0% probability); Augsburg, Hugo-Eckener-Str. gr. 5: double grave, combined calibration of both burials: 2469–2310 BC (95.4% probability) (Table 2). The three latest dates for BBP graves derive from the cemetery Hugo-Eckener-Str. (graves 1, 9 and 10) and are almost identical in their dating with the latest 2σ calibrated time span ending in 2039 BC (grave 1). All the other dates for BBP graves are evenly distributed between the oldest and the youngest date (Fig 3). The sequence of clusters of BBP dates in Fig 3 does not correspond with any archaeological division of the BBP, but only results from a sequence of small plateaus of the calibration curve.

**Table 2:** Burials from the Augsburg cemeteries and their radiocarbon dates (na = not analyzed).

Name of site	GraveNo.	GraveNo. after	Feat.No.	Ind.	LaborNo.	C14 Age	±	Cal 1 sigma (68,2% probability)	Cal 2 sigma (95,4% probability)	C:N	%C
Augsburg, Hugo-Eckener-Straße	1	Kociumaka	167		MAMS 18912	3741	24	cal BC 2199–2062	cal BC 2270–2039	3.3	36.9
Augsburg, Hugo-Eckener-Straße	3	Kociumaka	168		MAMS 18913	3788	23	cal BC 2280–2148	cal BC 2289–2141	3.3	37.3
Augsburg, Hugo-Eckener-Straße	5	Kociumaka	169	skeleton 1	MAMS 18914	3942	25	cal BC 2487–2350	cal BC 2562–2345	3.1	53.4
Augsburg, Hugo-Eckener-Straße	5	Kociumaka	169	skeleton 2	MAMS 18915	3863	25	cal BC 2453–2289	cal BC 2461–2211	3.3	53.1
Augsburg, Hugo-Eckener-Straße	6	Kociumaka	170		MAMS 18916	3774	24	cal BC 2274–2142	cal BC 2288–2066	3.3	43.2
Augsburg, Hugo-Eckener-Straße	7	Kociumaka	171		MAMS 18917	3815	25	cal BC 2289–2206	cal BC 2387–2146	3.3	30.4
Augsburg, Hugo-Eckener-Straße	8	Kociumaka	180	skeleton 1	MAMS 18918	3860	25	cal BC 2453–2287	cal BC 2461–2210	3.3	47.3
Augsburg, Hugo-Eckener-Straße	8	Kociumaka	180	skeleton 2	MAMS 18919	3871	25	cal BC 2453–2295	cal BC 2463–2235	3.3	45.1
Augsburg, Hugo-Eckener-Straße	9	Kociumaka	183		MAMS 18920	3743	19	cal BC 2199–2066	cal BC 2204–2043	3.3	53.7
Augsburg, Hugo-Eckener-Straße	10	Kociumaka	190		MAMS 18921	3748	19	cal BC 2199–2138	cal BC 2268–2046	3.3	47.8
Haunstetten, Postillionstraße	3	Massy	24		MAMS 18959	3679	20	cal BC 2131–2029	cal BC 2137–1980	3.3	45.5
Haunstetten, Postillionstraße	4	Massy	12		MAMS 18956	3697	20	cal BC 2134–2037	cal BC 2190–2028	3.3	54.3
Haunstetten, Postillionstraße	5	Massy	38		MAMS 18963	3717	23	cal BC 2191–2042	cal BC 2197–2034	3.3	52.7
Haunstetten, Postillionstraße	6	Massy	44		MAMS 18964	3681	23	cal BC 2131–2029	cal BC 2139–1979	3.1	49.0
Haunstetten, Postillionstraße	8	Massy	140		MAMS 18973	3631	20	cal BC 2024–1963	cal BC 2114–1926	3.3	47.0
Haunstetten, Postillionstraße	9	Massy	28		MAMS 18960	3608	20	cal BC 2016–1936	cal BC 2027–1906	3.3	35.5
Haunstetten, Postillionstraße	11	Massy	32		MAMS 18961	3619	20	cal BC 2020–1948	cal BC 2032–1916	3.3	45.4
Haunstetten, Postillionstraße	12	Massy	35		MAMS 18962	3621	20	cal BC 2021–1951	cal BC 2033–1918	3.3	44.5
Haunstetten, Postillionstraße	13	Massy	47		MAMS 18965	3662	24	cal BC 2126–1979	cal BC 2134–1956	3.3	54.7
Haunstetten, Postillionstraße	14	Massy	50		MAMS 18966	3707	24	cal BC 2138–2040	cal BC 2196–2029	3.3	48.5
Haunstetten, Postillionstraße	16	Massy	84		MAMS 18967	3638	24	cal BC 2030–1962	cal BC 2126–1928	3.3	49.1
Haunstetten, Postillionstraße	19	Massy	131		MAMS 18971	3635	20	cal BC 2027–1965	cal BC 2120–1938	3.3	51.3
Haunstetten, Postillionstraße	21	Massy	85		MAMS 18968	3631	24	cal BC 2026–1959	cal BC 2120–1921	3.3	46.9
Haunstetten, Postillionstraße	24	Massy	137		MAMS 18972	3613	20	cal BC 2017–1941	cal BC 2029–1912	3.3	43.9
Haunstetten, Postillionstraße	30	Massy	111		MAMS 18970	3639	20	cal BC 2031–1966	cal BC 2122–1941	3.3	51.7
Haunstetten, Postillionstraße	32	Massy	99		MAMS 18969	3641	25	cal BC 2033–1960	cal BC 2130–1930	3.3	40.0
Haunstetten, Postillionstraße	35	Massy	16		MAMS 18957	3612	20	cal BC 2018–1941	cal BC 2029–1911	3.3	41.6
Haunstetten, Postillionstraße	36	Massy	6		MAMS 18955	3574	19	cal BC 1942–1895	cal BC 2009–1883	3.3	49.0
Haunstetten, Postillionstraße	38	Massy	3		MAMS 18954	3592	19	cal BC 2006–1911	cal BC 2019–1890	3.3	52.0
Haunstetten, Postillionstraße	39	Massy	21		MAMS 18958	3606	20	cal BC 2014–1931	cal BC 2025–1903	3.1	47.0
Haunstetten, Postillionstraße	40	Massy	1		MAMS 18952	3583	28	cal BC 1964–1892	cal BC 2024–1882	3.3	49.5
Haunstetten, Postillionstraße	41	Massy	2		MAMS 18953	3648	19	cal BC 2111–1975	cal BC 2126–1948	3.4	54.5
Haunstetten, Unterer Talweg 58–62	3	Massy	153		MAMS 18943	3553	24	cal BC 1941–1836	cal BC 1971–1776	3.3	53.1
Haunstetten, Unterer Talweg 58–62	4	Massy	152	skeleton 1	MAMS 18942	3558	23	cal BC 1939–1885	cal BC 2007–1779	3.3	51.8
Haunstetten, Unterer Talweg 58–62	5	Massy	151		MAMS 18940	3568	24	cal BC 1943–1890	cal BC 2016–1782	3.1	35.5
Haunstetten, Unterer Talweg 58–62	5	Massy	151		MAMS 18941	3557	20	cal BC 1931–1886	cal BC 1961–1781	3.1	40.5
Haunstetten, Unterer Talweg 58–62	5	Massy	151		MAMS 18940+18941 combined	3566	11	cal BC 1930–1894	cal BC 1946–1886	na	na
Haunstetten, Unterer Talweg 58–62	6	Massy	150		MAMS 18939	3559	24	cal BC 1939–1885	cal BC 2009–1780	3.3	54.3
Haunstetten, Unterer Talweg 58–62	7	Kociumaka	67		MAMS 18934	3840	20	cal BC 2340–2211	cal BC 2455–2204	3.3	67.6
Haunstetten, Unterer Talweg 58–62	7	Massy	149		MAMS 18938	3597	24	cal BC 2011–1917	cal BC 2023–1892	3.3	47.9
Haunstetten, Unterer Talweg 58–62	8	Kociumaka	68	skeleton 2	MAMS 18935	3910	20	cal BC 2466–2349	cal BC 2470–2310	3.3	52.2
Haunstetten, Unterer Talweg 58–62	9	Massy	147		MAMS 18937	3612	25	cal BC 2020–1938	cal BC 2031–1900	3.3	53.8
Haunstetten, Unterer Talweg 58–62	10	Massy	146		MAMS 18933	3570	19	cal BC 1940–1894	cal BC 2009–1881	3.3	41.7
Haunstetten, Unterer Talweg 85	1	Massy	1163		MAMS 18946	3586	24	cal BC 1961–1895	cal BC 2021–1885	3.3	43.6
Haunstetten, Unterer Talweg 85	2	Massy	1410		MAMS 18950	3549	25	cal BC 1940–1829	cal BC 1960–1774	3.3	47.9
Haunstetten, Unterer Talweg 85	3	Massy	1412		MAMS 18951	3602	25	cal BC 2014–1923	cal BC 2025–1895	3.0	54.2
Haunstetten, Unterer Talweg 85	I/1	Kociumaka	1334		MAMS 18947	3827	25	cal BC 2331–2206	cal BC 2453–2152	3.2	26.1
Haunstetten, Unterer Talweg 85	I/2	Kociumaka	1336		MAMS 18948	3893	22	cal BC 2458–2347	cal BC 2465–2300	3.3	51.6
Haunstetten, Unterer Talweg 85	I/3	Kociumaka	1343		MAMS 18949	3819	24	cal BC 2291–2206	cal BC 2397–2149	3.3	47.5
Haunstetten, Unterer Talweg 85	II/1	Kociumaka	113		MAMS 18945	3831	24	cal BC 2334–2207	cal BC 2456–2155	3.1	52.3
Haunstetten, Unterer Talweg 85	II/2	Kociumaka	110		MAMS 18944	3789	24	cal BC 2281–2149	cal BC 2290–2141	3.3	43.0
Kleinaitingen, Gewerbegebiet Nord	2	Massy	3		MAMS 21563	3616	28	cal BC 2021–1942	cal BC 2111–1895	3.3	38.9
Kleinaitingen, Gewerbegebiet Nord	3	Massy	2		MAMS 21562	3477	28	cal BC 1876–1750	cal BC 1884–1698	3.3	23.9
Kleinaitingen, Gewerbegebiet Nord	4	Massy	5		MAMS 21564	3493	28	cal BC 1878–1771	cal BC 1892–1704	3.3	20.9
Kleinaitingen, Gewerbegebiet Nord	6	Massy	37		MAMS 21569	3560	28	cal BC 1946–1883	cal BC 2013–1777	3.3	36.9
Kleinaitingen, Gewerbegebiet Nord	9	Massy	33		MAMS 21566	3364	28	cal BC 1687–1626	cal BC 1742–1562	3.1	39.8
Kleinaitingen, Gewerbegebiet Nord	10	Massy	40		MAMS 21570	3469	28	cal BC 1875–1705	cal BC 1882–1695	3.3	13.3
Kleinaitingen, Gewerbegebiet Nord	11	Massy	35		MAMS 21567	3582	28	cal BC 1962–1891	cal BC 2026–1880	3.3	38.3
Kleinaitingen, Gewerbegebiet Nord	12	Massy	32		MAMS 21565	3454	28	cal BC 1871–1696	cal BC 1878–1691	na	na
Kleinaitingen, Gewerbegebiet Nord	13	Massy	50		MAMS 21572	3505	33	cal BC 1884–1773	cal BC 1920–1705	3.3	38.9
Kleinaitingen, Gewerbegebiet Nord	14	Massy	36		MAMS 21568	3552	27	cal BC 1943–1830	cal BC 2006–1774	3.3	40.6
Kleinaitingen, Gewerbegebiet Nord	15	Massy	51		MAMS 21573	3531	34	cal BC 1919–1777	cal BC 1946–1754	3.1	39.4
Kleinaitingen, Gewerbegebiet Nord	17	Massy	53		MAMS 21574	3492	33	cal BC 1878–1770	cal BC 1901–1698	3.1	39.6
Kleinaitingen, Gewerbegebiet Nord	21	Massy	43		MAMS 21571	3486	27	cal BC 1877–1761	cal BC 1888–1701	3.1	30.4
Kleinaitingen, Gewerbegebiet Nord	22	Massy	55		MAMS 21575	3504	33	cal BC 1884–1773	cal BC 1919–1705	na	18.8
Kleinaitingen, Gewerbegebiet Nord	23	Massy	69		MAMS 21581	3294	34	cal BC 1613–1531	cal BC 1657–1500	3.4	17.4
Kleinaitingen, Gewerbegebiet Nord	24	Massy	62	skeleton A	MAMS 21576	3459	34	cal BC 1874–1697	cal BC 1881–1691	2.5	30.2
Kleinaitingen, Gewerbegebiet Nord	24	Massy	62	skeleton B	MAMS 21577	3478	33	cal BC 1877–1749	cal BC 1889–1695	3.4	27.2
Kleinaitingen, Gewerbegebiet Nord	26	Massy	66		MAMS 21580	3489	34	cal BC 1878–1767	cal BC 1899–1696	3.3	40.9
Kleinaitingen, Gewerbegebiet Nord	28	Massy	77	skeleton A	MAMS 21584	3548	34	cal BC 1943–1782	cal BC 2008–1769	na	na
Kleinaitingen, Gewerbegebiet Nord	28	Massy	77	skeleton B	MAMS 21585	3469	35	cal BC 1876–1702	cal BC 1885–1693	3.1	38.3
Kleinaitingen, Gewerbegebiet Nord	29	Massy	78		MAMS 21586	3474	34	cal BC 1877–1746	cal BC 1887–1694	na	22.0
Kleinaitingen, Gewerbegebiet Nord	34	Massy	87		MAMS 21588	3433	28	cal BC 1767–1690	cal BC 1876–1660	3.4	23.8
Kleinaitingen, Gewerbegebiet Nord	35	Massy	86		MAMS 21587	3480	34	cal BC 1877–1750	cal BC 1891–1694	na	na
Kleinaitingen, Gewerbegebiet Nord	37	Massy	119		MAMS 21594	3470	27	cal BC 1876–1745	cal BC 1882–1696	3.3	24.0
Kleinaitingen, Gewerbegebiet Nord	38	Massy	120		MAMS 21595	3417	27	cal BC 1748–1684	cal BC 1866–1638	3.4	14.9
Kleinaitingen, Gewerbegebiet Nord	40	Massy	72		MAMS 21583	3508	34	cal BC 1886–1773	cal BC 1926–1705	3.3	19.5
Kleinaitingen, Gewerbegebiet Nord	41	Massy	70		MAMS 21582	3594	37	cal BC 2013–1899	cal BC 2116–1785	3.1	39.5
Kleinaitingen, Gewerbegebiet Nord	43	Massy	65	adult	MAMS 21578	3456	34	cal BC 1873–1695	cal BC 1881–1690	3.4	33.0
Kleinaitingen, Gewerbegebiet Nord	43	Massy	65	child	MAMS 21579	3329	35	cal BC 1660–1536	cal BC 1690–1514	3.4	38.4
Kleinaitingen, Gewerbegebiet Nord	47	Massy	98		MAMS 21592	3370	27	cal BC 1688–1629	cal BC 1742–1613	3.3	16.0
Kleinaitingen, Gewerbegebiet Nord	48	Massy	92		MAMS 21590	3489	28	cal BC 1878–1768	cal BC 1889–1701	3.3	21.5
Kleinaitingen, Gewerbegebiet Nord	56	Massy	114		MAMS 21593	3563	28	cal BC 1948–1883	cal BC 2015–1778	na	18.3
Kleinaitingen, Gewerbegebiet Nord	60	Massy	95		MAMS 21591	3422	28	cal BC 1754–1685	cal BC 1870–1640	na	15.6
Kleinaitingen, Gewerbegebiet Nord	62	Massy	89		MAMS 21589	3292	28	cal BC 1611–1532	cal BC 1627–1505	3.3	22.6
Königsbrunn, Ampack	1	Kociumaka	none		MAMS 18887	3924	23	cal BC 2471–2350	cal BC 2476–2310	3.2	47.8
Königsbrunn, Obere Kreuzstraße	2	Massy	96		MAMS 18910	3567	23	cal BC 1942–1889	cal BC 2013–1784	3.3	53.6
Königsbrunn, Obere Kreuzstraße	3	Massy	47		MAMS 18894	3671	22	cal BC 2129–1983	cal BC 2136–1977	3.2	48.4
Königsbrunn, Obere Kreuzstraße	6	Massy	2		MAMS 18888	3599	22	cal BC 2011–1920	cal BC 2023–1894	3.2	48.7
Königsbrunn, Obere Kreuzstraße	9	Massy	95		MAMS 18909	3608	23	cal BC 2016–1931	cal BC 2027–1901	3.3	54.1
Königsbrunn, Obere Kreuzstraße	14	Massy	5		MAMS 18889	3626	22	cal BC 2023–1954	cal BC 2113–1917	3.2	38.5
Königsbrunn, Obere Kreuzstraße	15	Massy	6		MAMS 18890	3611	23	cal BC 2019–1938	cal BC 2029–1903	3.2	42.6
Königsbrunn, Obere Kreuzstraße	17	Massy	50		MAMS 18895	3623	23	cal BC 2022–1951	cal BC 2112–1912	3.2	48.1
Königsbrunn, Obere Kreuzstraße	23	Massy	80		MAMS 18901	3664	24	cal BC 2127–1980	cal BC 2134–1959	3.1	53.5
Königsbrunn, Obere Kreuzstraße	24	Massy	66		MAMS 18896	3609	22	cal BC 2018–1936	cal BC 2028–1903	3.2	44.8
Königsbrunn, Obere Kreuzstraße	25	Massy	67		MAMS 18897	3599	22	cal BC 2011–1920	cal BC 2023–1894	3.3	53.9
Königsbrunn, Obere Kreuzstraße	29	Massy	93		MAMS 18908	3621	23	cal BC 2022–1948	cal BC 2111–1907	3.3	54.4
Königsbrunn, Obere Kreuzstraße	31	Massy	76		MAMS 18899	3567	23	cal BC 1941–1889	cal BC 2013–1785	3.3	52.3
Königsbrunn, Obere Kreuzstraße	33	Massy	9	skeleton A	MAMS 18891	3596	22	cal BC 2010–1918	cal BC 2022–1892	3.2	47.1
Königsbrunn, Obere Kreuzstraße	33	Massy	9	skeleton B	MAMS 18892	3616	23	cal BC 2021–1943	cal BC 2032–1903	3.2	47.2
Königsbrunn, Obere Kreuzstraße	35	Massy	84		MAMS 18905	3575	25	cal BC 1949–1891	cal BC 2021–1830	3.1	52.6
Königsbrunn, Obere Kreuzstraße	36	Massy	73		MAMS 18898	3603	23	cal BC 2013–1924	cal BC 2024–1897	3.1	54.6
Königsbrunn, Obere Kreuzstraße	38	Massy	83		MAMS 18904	3562	24	cal BC 1941–1886	cal BC 2011–1780	3.3	52.1
Königsbrunn, Obere Kreuzstraße	39	Massy	82		MAMS 18903	3600	24	cal BC 2012–1921	cal BC 2023–1894	3.3	53.0
Königsbrunn, Obere Kreuzstraße	40	Massy	81		MAMS 18902	3602	24	cal BC 2013–1923	cal BC 2024–1896	3.3	53.4
Königsbrunn, Obere Kreuzstraße	43	Massy	86		MAMS 18907	3615	24	cal BC 2021–1942	cal BC 2032–1901	3.3	48.6
Königsbrunn, Obere Kreuzstraße	45	Massy	79		MAMS 18900	3581	23	cal BC 1953–1894	cal BC 2018–1883	3.3	52.6
Königsbrunn, Obere Kreuzstraße	46	Massy	85		MAMS 18906	3625	23	cal BC 2023–1953	cal BC 2113–1915	3.3	53.8
Königsbrunn, Obere Kreuzstraße	47	Massy	117		MAMS 18911	3581	23	cal BC 1953–1894	cal BC 2019–1883	3.3	54.1
Königsbrunn, Obere Kreuzstraße	48	Massy	26		MAMS 18893	3618	22	cal BC 2021–1946	cal BC 2033–1907	3.2	40.8
Oberottmarshausen, Kiesgrube Lauter	1	Massy	87		MAMS 21544	3324	34	cal BC 1643–1534	cal BC 1687–1517	3.3	32.6
Oberottmarshausen, Kiesgrube Lauter	2	Massy	92		MAMS 21546	3132	42	cal BC 1449–1308	cal BC 1497–1293	3.3	37.9
Oberottmarshausen, Kiesgrube Lauter	3	Massy	91		MAMS 21545	3075	41	cal BC 1399–1289	cal BC 1426–1230	3.4	9.7
Oberottmarshausen, Kiesgrube Lauter	5	Massy	109		MAMS 21550	3360	37	cal BC 1728–1615	cal BC 1743–1534	3.3	19.9
Oberottmarshausen, Kiesgrube Lauter	7	Massy	110		MAMS 21551	3207	37	cal BC 1504–1437	cal BC 1605–1412	3.3	14.7
Oberottmarshausen, Kiesgrube Lauter	9	Massy	100		MAMS 21549	3225	36	cal BC 1526–1448	cal BC 1608–1427	3.3	10.7
Oberottmarshausen, Kiesgrube Lauter	12	Massy	94		MAMS 21547	3124	36	cal BC 1435–1309	cal BC 1493–1287	3.3	12.9
Oberottmarshausen, Kiesgrube Lauter	14	Massy	79		MAMS 21541	3227	33	cal BC 1526–1450	cal BC 1608–1430	3.0	37.2
Oberottmarshausen, Kiesgrube Lauter	17	Massy	84		MAMS 21543	3283	32	cal BC 1610–1527	cal BC 1636–1465	3.3	38.8
Oberottmarshausen, Kiesgrube Lauter	18	Massy	97		MAMS 21548	3310	37	cal BC 1627–1531	cal BC 1682–1505	3.3	40.5
Oberottmarshausen, Kiesgrube Lauter	19	Massy	81		MAMS 21542	3277	33	cal BC 1609–1511	cal BC 1630–1459	na	22.3
Oberottmarshausen, Kiesgrube Lauter	21	Massy	142		MAMS 21554	3309	37	cal BC 1625–1531	cal BC 1682–1504	3.3	14.6
Oberottmarshausen, Kiesgrube Lauter	25	Massy	151	skeleton 1	MAMS 21555	3370	38	cal BC 1732–1623	cal BC 1749–1535	3.1	38.9
Oberottmarshausen, Kiesgrube Lauter	25	Massy	151	skeleton 2	MAMS 21556	3288	27	cal BC 1610–1530	cal BC 1623–1504	3.3	38.1
Oberottmarshausen, Kiesgrube Lauter	26	Massy	141	adult	MAMS 21552	3297	36	cal BC 1616–1530	cal BC 1681–1499	3.3	9.8
Oberottmarshausen, Kiesgrube Lauter	26	Massy	141	child	MAMS 21553	3296	36	cal BC 1615–1530	cal BC 1664–1498	3.3	37.9
Oberottmarshausen, Kiesgrube Lauter	27	Massy	154		MAMS 21558	3449	72	cal BC 1878–1687	cal BC 1949–1563	3.3	37.2
Oberottmarshausen, Kiesgrube Lauter	28	Massy	152		MAMS 21557	3295	28	cal BC 1612–1532	cal BC 1630–1505	3.3	38.1
Oberottmarshausen, Kiesgrube Lauter	29	Massy	162		MAMS 21560	3282	28	cal BC 1610–1527	cal BC 1622–1502	3.3	38.7
Oberottmarshausen, Kiesgrube Lauter	30	Massy	165		MAMS 21561	3349	29	cal BC 1683–1616	cal BC 1735–1535	3.3	38.1
Oberottmarshausen, Kiesgrube Lauter	31	Massy	156		MAMS 21559	3241	28	cal BC 1597–1455	cal BC 1609–1442	3.3	38.7
Wehringen, Hochfeld	2	Massy	1375		MAMS 18925	3555	19	cal BC 1930–1885	cal BC 1953–1781	3.3	42.9
Wehringen, Hochfeld	3	Massy	1564		MAMS 18931	3594	19	cal BC 2007–1917	cal BC 2020–1892	3.3	53.9
Wehringen, Hochfeld	4	Massy	1415	child	MAMS 18928	3596	19	cal BC 2008–1919	cal BC 2021–1893	3.3	54.1
Wehringen, Hochfeld	4	Massy	1415	adult	MAMS 18929	3573	19	cal BC 1941–1895	cal BC 2008–1883	3.3	40.9
Wehringen, Hochfeld	5	Massy	1414		MAMS 18927	3601	19	cal BC 2011–1926	cal BC 2022–1897	3.0	43.0
Wehringen, Hochfeld	6	Massy	1380		MAMS 18926	3598	18	cal BC 2009–1922	cal BC 2021–1895	3.3	38.1
Wehringen, Hochfeld	7	Massy	1474		MAMS 18930	3614	19	cal BC 2017–1943	cal BC 2029–1916	3.1	54.5
Wehringen, Hochfeld	8	Massy	1586		MAMS 18932	3550	19	cal BC 1932–1881	cal BC 1951–1779	3.3	53.7
Wehringen, Hochfeld	13	Massy	1193		MAMS 18924	3544	19	cal BC 1927–1830	cal BC 1945–1777	3.1	35.0
Wehringen, Hochfeld	14	Massy	1192	skeleton A	MAMS 18922	3810	19	cal BC 2285–2206	cal BC 2331–2150	3.3	54.1
Wehringen, Hochfeld	14	Massy	1192	skeleton B	MAMS 18923	3523	18	cal BC 1892–1779	cal BC 1918–1772	3.3	40.1

**Fig 4:**
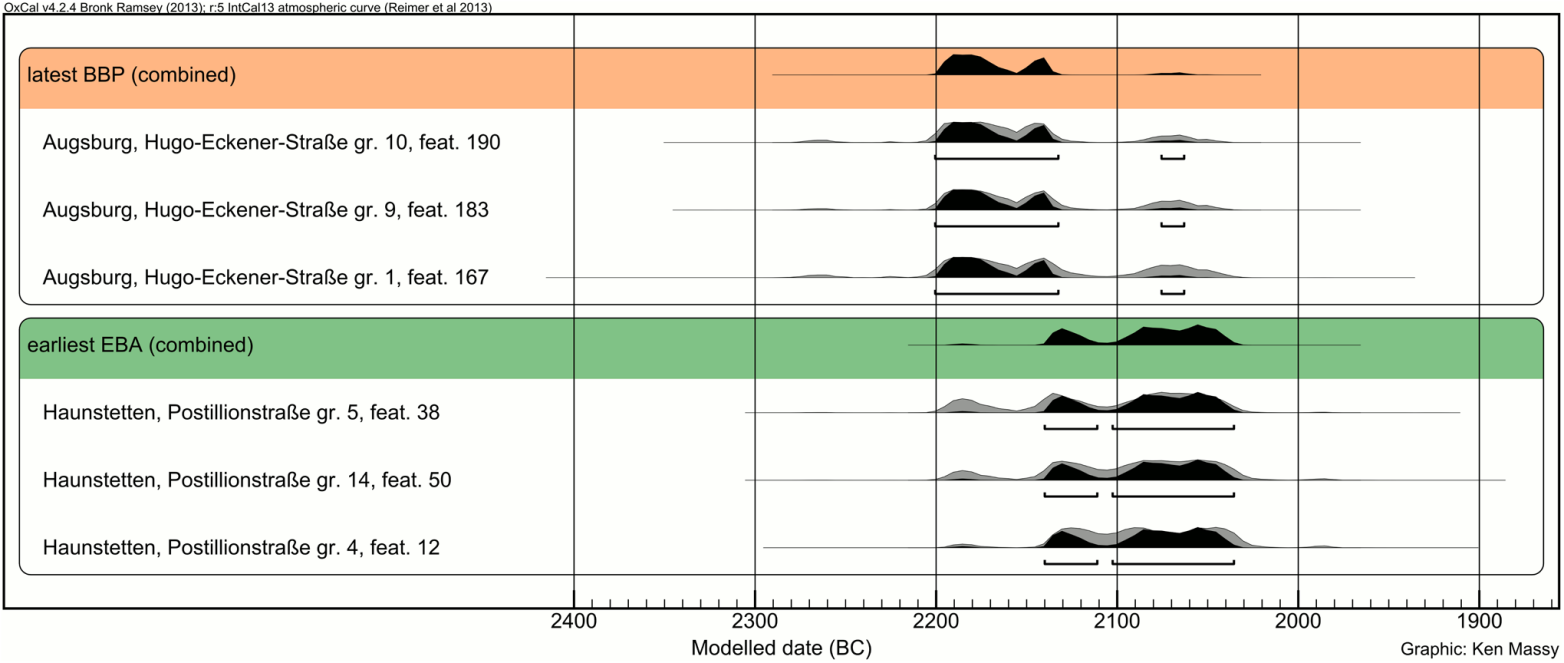
Combined calibration of the radiocarbon dates from the latest BBP and earliest EBA burials from the Augsburg region.

At first, it seems difficult to decide whether there is a significant overlap between the latest BBP and the earliest EBA burials. If one supposes that the three latest BBP burials were buried at almost the same time (the three related uncalibrated dates fall within a range of seven years: 3748–3741 ^14^C BP), one can calculate a combined calibrated age, which significantly narrows the time range with the highest probability: 2201–2133 BC (93.0% probability) and 2076–2064 BC (2.4% probability). This indicates that the last BBP burials in the Augsburg region were most probably not laid down after the early second half of the 22^nd^ century (Fig 4).

The oldest EBA dates all derive from the cemetery Haunstetten, Postillonstraße, namely graves 4, 5 and 14 (the three related uncalibrated dates fall within a range of 20 years: 3717–3697 ^14^C BP). The narrow time range of their deposition makes it possible to calculate a combined calibrated age. The 2σ calibrated range spans 2141–2112 BC (22.8% probability) and 2103–2036 BC (72.6% probability) BC.

**Fig 5:**
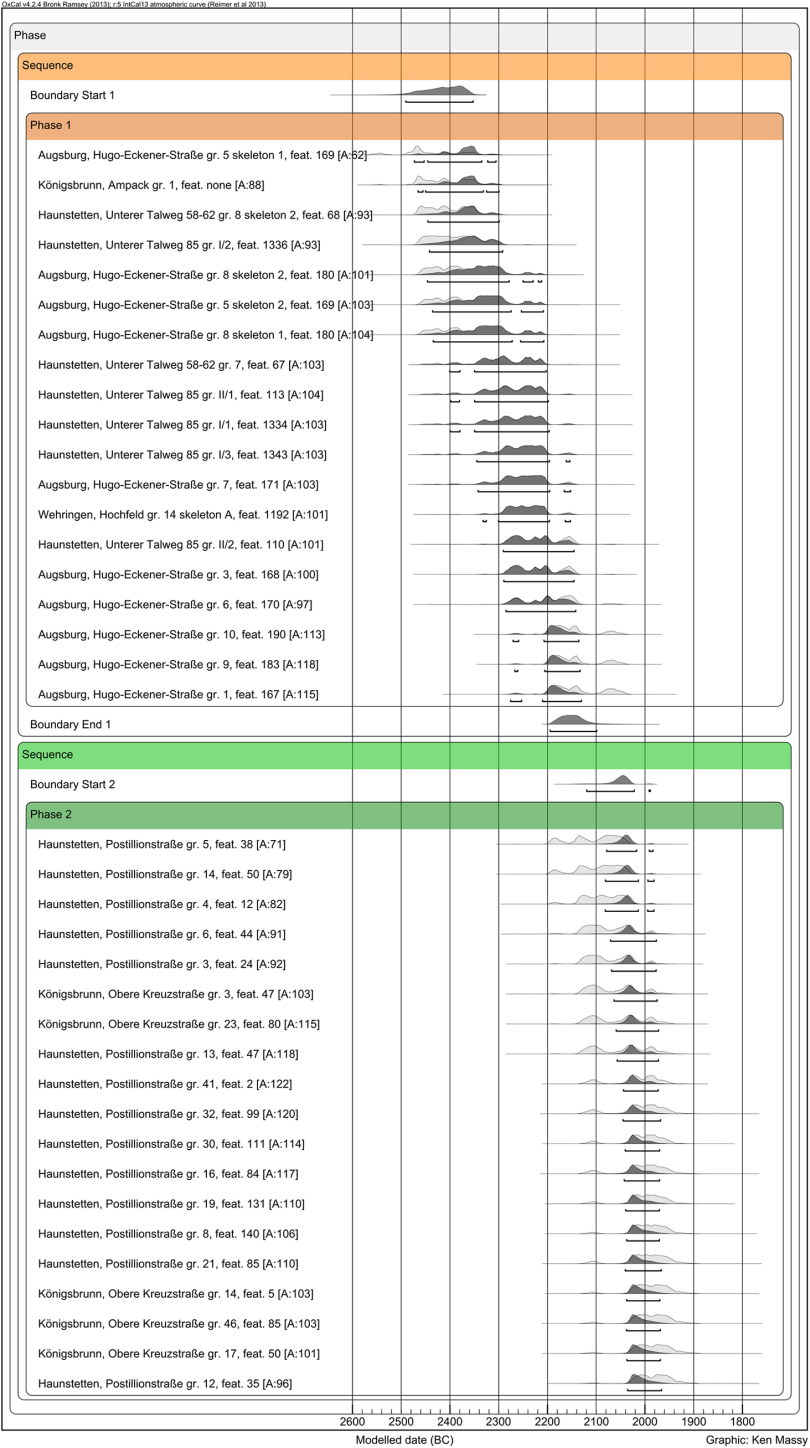
Bayesian modeling of the BBP/EBA overlap on the basis of all 19 BBP and the 19 oldest EBA burials. The Agreement Index [A:] lies over 60% within every measurement.

Calculating the overlap of the two phases (BBP and EBA) using a multi-phase model in Oxcal v4.2.24 [36] with IntCal13 [37] strengthens the argument of a sequence (Fig 5). Modeled values of “Boundary End 1” (end of BBP) and “Boundary Start 2” (beginning of EBA) do not overlap more than 20 years.

Therefore, one can assume that there is hardly any overlap or no overlap at all between the latest BBP and the earliest EBA burials. At least in the Augsburg region, there is definitely no indication for either a substantial overlap or a hiatus between both periods. The data suggest a continuous and fluent sequence from the BBP to the EBA.

The new dates also help to better understand the development of the EBA itself. We are able to connect the traditional division of Bz A into the sub-phases Bz A1 and A2 with absolute dates for the respective type objects. Ruckdeschel defined the following pin types as type objects for Bz A1 (with its sub-phases Bz A1a and Bz A1b) and Bz A2 (with its sub-phases Bz A2a, Bz A2b and Bz A2c) [15] (see below):

Bz A1a: *Ruderkopfnadeln* (with large or small head), bone pins and boar tusk pins
Bz A1a–A1b: *Scheibenkopfnadeln*
Bz A1b: *Schleifenkopfnadeln* and *Horkheimer Nadeln*.
Bz A2a: *Ösenkopfnadel*, *Hülsenkopfnadel* and *schräg durchlochte Kugelkopfnadel*.

We were not able to date graves with Bz A2b and Bz A2c type objects, which are very rare in southern Germany (cf. [17]). The *Lochhalsnadel* type *Paarstadl* was dated by Ruckdeschel to Bz A2c, but it has meanwhile been dated to the beginning of the Middle Bronze Age (Bz B) by other authors [45], [17]. We agree with the Bz B date for this type of pin, also because the specimen dated by us was associated with a most characteristic Bz B dagger in one of the burials (Oberottmarshausen gr. 5). Therefore, we see the following pin types from our grave inventories as characteristic of Bz B: *Lochhalsnadel* type *Paarstadl* and *Dreiringnadel* type *Muschenheim*. *Rollenkopfnadeln mit tordiertem Schaft* start in Bz B, but continue well into the later MBA [46].

**Fig 6:**
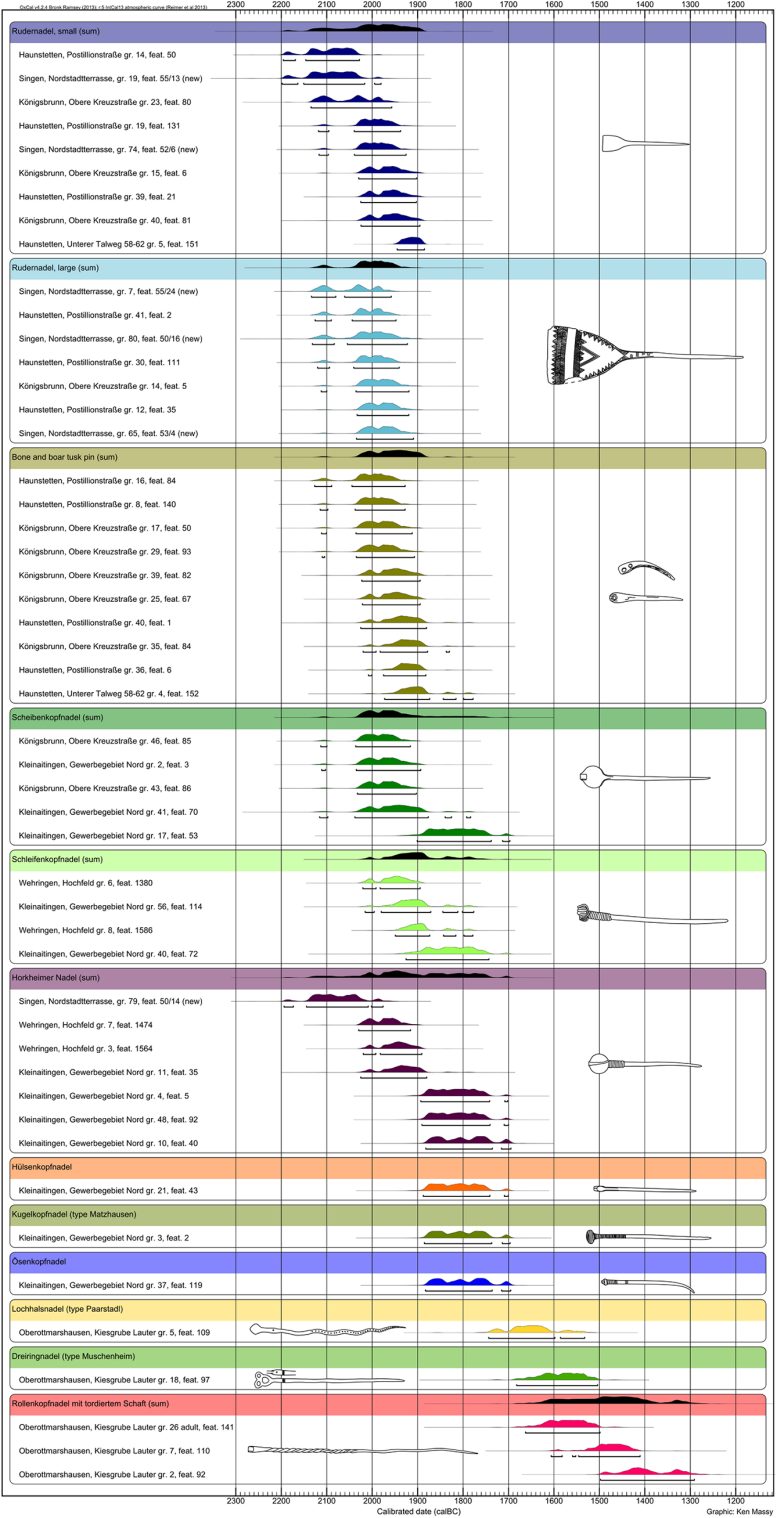
Plot of all pins with radiocarbon dates from Singen and the Augsburg region. Pin types with more than two dates are supplemented with a sum calibration (black) to show their overall timespan.

We could date altogether 36 graves with Bz A1 pins, three graves with Bz A2 pins and five graves with MBA pin types (Fig 6).

Graves with Bz A1a pins are among the oldest EBA graves analyzed within our project (Haunstetten, Postillonstr. gr. 14). Several 2σ calibrated ranges start shortly after 2200 BC, but the 2σ calibrated range of most of these graves spans the second half of the 21^st^ century and the complete 20^th^ century BC. Therefore, we propose a period of deposition of these pins from ca 2150/2100 BC until 1900 BC for the Augsburg region. The 2σ calibrated ranges of the two type objects of Bz A1b start in the late 21^st^ century BC (Wehringen, Hochfeld grave 7: 2030–1916 BC with 95.4% probability). However, these pin types have a much longer period of use and were still deposited after 1900 BC and probably even until 1700 BC (e.g. Kleinaitingen, Gewerbegebiet Nord grave 10: 1883–1736 BC with 87.5% probability and 1716–1695 BC with 7.9% probability). It is clear that Bz A1a and Bz A1b are contemporaneous for around 150 years. Taken together, Bz A1 can be dated in the Augsburg region to the centuries between 2150/2100 and 1750/1700 BC.

**Fig 7:**
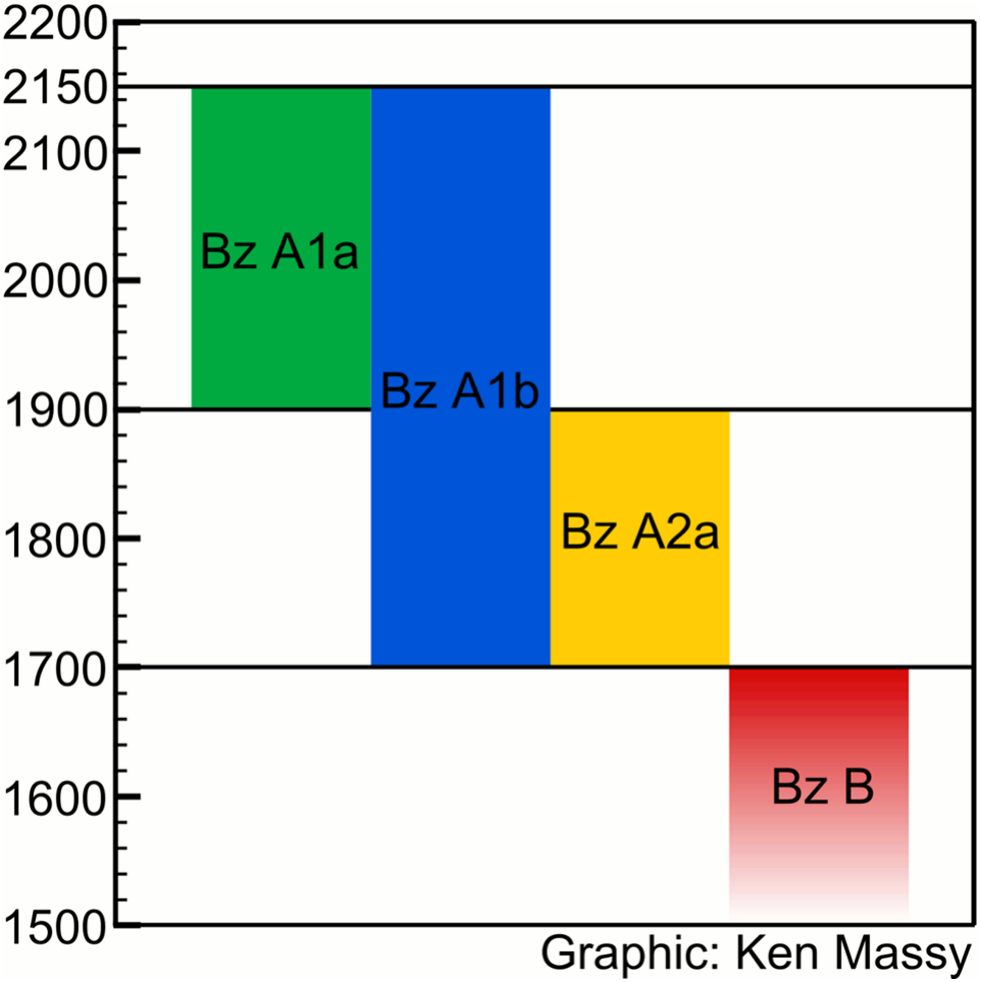
Chronological sketch based on the radiocarbon dates from pins of Singen and Augsburg.

The three dates with classical Bz A2 pin types (i.e. Bz A2a in the Ruckdeschel system)–all from Kleinaitingen–were extremely similar with almost the same uncalibrated dates and indicate a deposition between ca 1900 BC and the years around 1700 BC. Compared with the Bz A1 dates, it seems that Bz A2 pins were worn and deposited contemporaneously with Bz A1b pins, whereas Bz A1a pins could have been replaced by Bz A2 types (Fig 7).

The oldest burial with type objects of Bz B (the first phase of the MBA) is Oberottmarshausen, grave 5 (1744–1598 BC with 84.0% probability and 1586–1533 BC with 11.4%probability). It contains a *Lochhalsnadel* type *Paarstadl* and a dagger with trapezoid hilt (i.e. [47]: series K34 or Q60). Therefore, it is most probable that the deposition of clear Bz B type objects started already in the 17^th^ century BC in the Augsburg region. The other grave with a Bz B pin type, i.e. Oberottmarshausen grave 18, dates to the 17^th^ or 16^th^ century BC. However, the small number of graves with Bz B type objects does not allow further insights into the chronological position and duration of Bz B. Unfortunately, the *Rollenkopfnadeln mit tordiertem Schaft* are not of typological significance, as they start in Bz B (and in Switzerland even late in Bz A2; cf. [46]) and continue further into the MBA.

**Fig 8:**
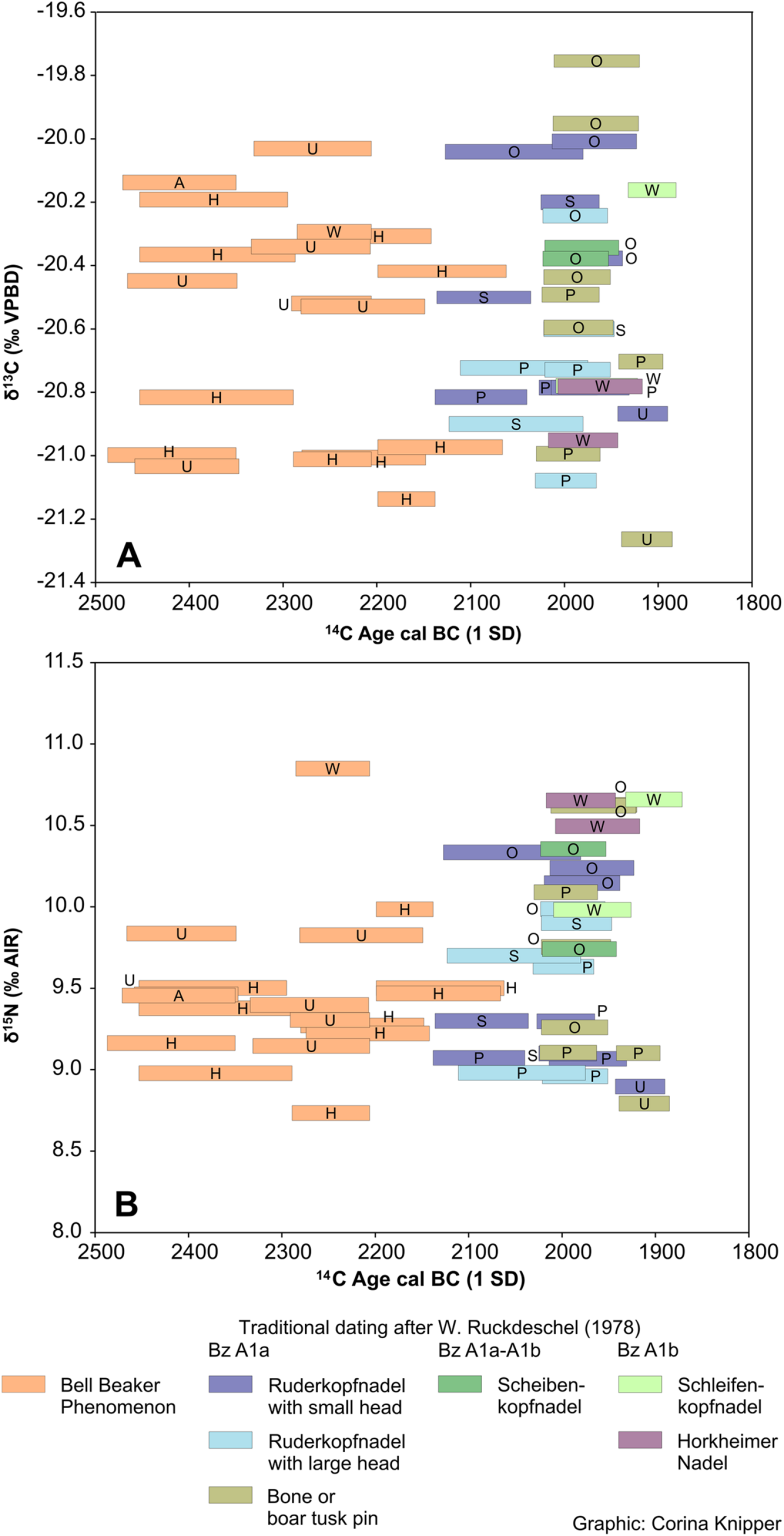
Plot of the calibrated radiocarbon dates against the δ^13^C values (A) and δ^15^N (B) values of the same collagen extracts. The letters indicate the burial sites: A: Königsbrunn "Ampack", H: Haunstetten "Hugo-Eckener-Strasse", O: Königsbrunn "Obere Kreuzstrasse", P: Haunstetten "Postillonsstrasse", U: Haunstetten "Unterer Talweg", W: Wehringen "Hochfeld". The C and N isotope data for Singen (S) are adopted from [42], [43].

Considerations about the absolute dating of relative chronological phases and periods of depositions of diagnostic artifacts need to consider reservoir effects, which may have shifted the radiocarbon ages by several hundred years (Table 3). Overall, the ^14^C data of the bone collagen of the BBP burials and especially those who were associated with certain EBA pin types appear comparatively consistent [48], [49]. They lack outliers towards older dates, which may have been caused by remarkable consumption of marine or freshwater food sources. This is in agreement with the stable carbon and nitrogen isotope ratios of bone collagen (Table 2). Despite the proximity of the Lech and Wertach Rivers, the δ^13^C values of between -21.3 and -19.8 ‰ and δ^15^N values of between 8.7 and 10.8 ‰ point to a predominance of terrestrial food sources, including crops as well as meat and dairy products of domestic animals. As to be expected for the inland location of the study area, there is no indication of any contribution of marine food sources. The data are also largely in agreement with previously investigated Final Neolithic to Early Bronze Age burials from southern Bavaria for which fish consumption was essentially excluded [50], [51]. Fig 8A illustrates the wide overlaps of the δ^13^C data of individuals of the BBP and those buried with diagnostic EBA pins (Fig 8). There is no correlation between old ^14^C ages and high δ^15^N or low δ^13^C values, which would indicate freshwater fish consumption [52]. The δ^15^N values of the individuals of the EBA shown in Fig 8B are more variable (range: 8.8–10.7 ‰) than those of the BBP which concentrate between comparatively low values of 8.7 and 10.0 ‰ (Fig 8). This pattern indicates that the older radiocarbon ages of the BBP burials are indeed due to their earlier calendaric age instead of resulting from extensive freshwater fish consumption. The light stable isotope ratios and ^14^C ages of the individuals buried with diagnostic Bz A1a and A1b pins overlap widely. Apparently independent of the ^14^C ages and grave goods, the δ^15^N and δ^13^C values tend to cluster by sites. Despite the very similar environmental conditions, this points to community-specific proportions of plant and animal-derived food and/or landuse and soil management, such as manuring [53], [54]. Overall, the light stable isotope ratios and the spread of the radiocarbon dates do not hint on possible overestimations of the periods of deposition of certain pin types due to different extents of reservoir effects among the investigated individuals.

**Table 3:** Light stable isotope data of the burials of the BBP and those with diagnostic artifacts of the EBA. The data for the burials from Singen are adopted from [43].

Name of site	GraveNo.	GraveNo. after	Feat.No.	Archaeologial dating	Diagnostic artifact	%oll	N%	C%	C/N atom	δ13C (‰VPBD)	δ15N (‰AIR)
**Augsburg, Hugo-Eckener-Straße**	**1**	**Kociumaka**	**167**	**BBP**		**1.9**	**11.3**	**31.0**	**3.2**	**-20.4**	**9.5**
**Augsburg, Hugo-Eckener-Straße**	**3**	**Kociumaka**	**168**	**BBP**		**1.2**	**11.2**	**30.9**	**3.2**	**-21.0**	**9.3**
**Augsburg, Hugo-Eckener-Straße**	**5**	**Kociumaka**	**169**	**BBP**		**1.1**	**16.5**	**44.0**	**3.1**	**-21.0**	**9.2**
**Augsburg, Hugo-Eckener-Straße**	**5**	**Kociumaka**	**169**	**BBP**		**1.6**	**16.4**	**43.9**	**3.1**	**-20.8**	**9.0**
**Augsburg, Hugo-Eckener-Straße**	**6**	**Kociumaka**	**170**	**BBP**		**2.4**	**13.3**	**36.4**	**3.2**	**-20.3**	**9.2**
**Augsburg, Hugo-Eckener-Straße**	**7**	**Kociumaka**	**171**	**BBP**		**0.9**	**9.9**	**27.4**	**3.3**	**-21.0**	**8.7**
**Augsburg, Hugo-Eckener-Straße**	**8**	**Kociumaka**	**180**	**BBP**		**1.6**	**14.3**	**38.7**	**3.1**	**-20.4**	**9.4**
**Augsburg, Hugo-Eckener-Straße**	**8**	**Kociumaka**	**180**	**BBP**		**1.3**	**14.0**	**37.7**	**3.1**	**-20.2**	**9.5**
**Augsburg, Hugo-Eckener-Straße**	**9**	**Kociumaka**	**183**	**BBP**		**2.1**	**16.4**	**44.3**	**3.2**	**-21.0**	**9.5**
**Augsburg, Hugo-Eckener-Straße**	**10**	**Kociumaka**	**190**	**BBP**		**2.0**	**14.4**	**39.4**	**3.2**	**-21.1**	**10.0**
**Haunstetten, Unterer Talweg 58–62**	**8**	**Kociumaka**	**68**	**BBP**		**2.1**	**16.0**	**43.9**	**3.2**	**-20.4**	**9.8**
**Haunstetten, Unterer Talweg 85**	**I/1**	**Kociumaka**	**1334**	**BBP**		**3.1**	**11.1**	**30.5**	**3.2**	**-20.0**	**9.1**
**Haunstetten, Unterer Talweg 85**	**I/2**	**Kociumaka**	**1336**	**BBP**		**3.3**	**16.0**	**40.2**	**2.9**	**-21.0**	**9.5**
**Haunstetten, Unterer Talweg 85**	**I/3**	**Kociumaka**	**1343**	**BBP**		**2.7**	**15.0**	**38.3**	**3.0**	**-20.5**	**9.3**
**Haunstetten, Unterer Talweg 85**	**II/1**	**Kociumaka**	**113**	**BBP**		**2.6**	**16.8**	**46.0**	**3.2**	**-20.3**	**9.4**
**Haunstetten, Unterer Talweg 85**	**II/2**	**Kociumaka**	**110**	**BBP**		**1.4**	**13.3**	**36.5**	**3.2**	**-20.5**	**9.8**
**Königsbrunn, Ampack**	**1**	**Kociumaka**	**none**	**BBP**		**1.6**	**16.7**	**45.2**	**3.2**	**-20.1**	**9.5**
**Wehringen, Hochfeld**	**14 Ind. A**	**Massy**	**1192**	**BBP**		**2.8**	**16.8**	**46.0**	**3.2**	**-20.29**	**10.85**
**Haunstetten, Postillionstraße**	**14**	**Massy**	**50**	**EBA**	**Rudernadel w/ small head**	**1.5**	**15.6**	**39.7**	**3.0**	**-20.8**	**9.1**
**Haunstetten, Postillionstraße**	**19**	**Massy**	**131**	**EBA**	**Rudernadel w/ small head**	**2.3**	**16.4**	**43.6**	**3.1**	**-20.8**	**9.3**
**Haunstetten, Postillionstraße**	**39**	**Massy**	**21**	**EBA**	**Rudernadel w/ small head**	**2.7**	**15.4**	**39.2**	**3.0**	**-20.8**	**9.1**
**Haunstetten, Unterer Talweg 58–62**	**5**	**Massy**	**151**	**EBA**	**Rudernadel w/ small head**	**2.5**	**13.7**	**37.7**	**3.2**	**-20.9**	**8.9**
**Königsbrunn, Obere Kreuzstraße**	**15**	**Massy**	**6**	**EBA**	**Rudernadel w/ small head**	**2.2**	**15.6**	**39.5**	**3.2**	**-20.4**	**10.1**
**Königsbrunn, Obere Kreuzstraße**	**23**	**Massy**	**80**	**EBA**	**Rudernadel w/ small head**	**3.9**	**16.8**	**45.3**	**3.1**	**-20.0**	**10.3**
**Königsbrunn, Obere Kreuzstraße**	**40**	**Massy**	**81**	**EBA**	**Rudernadel w/ small head**	**4.3**	**16.7**	**45.3**	**3.1**	**-20.0**	**10.2**
**Singen, Nordstadtterrasse, Lessingstrasse**	**19**	**Krause**	**55/13**	**EBA**	**Rudernadel w/ small head**	**2.7**	**15.5**	**41.4**	**3.1**	**-20.5**	**9.3**
**Singen, Nordstadtterrasse, Widerholtstrasse**	**74**	**Krause**	**52/6**	**EBA**	**Rudernadel w/ small head**	**8.6**	**13.6**	**37.4**	**3.2**	**-20.2**	**9.1**
**Haunstetten, Postillionstraße**	**12**	**Massy**	**35**	**EBA**	**Rudernadel w/ large head**	**3.1**	**14.6**	**37.2**	**3.0**	**-20.7**	**9.0**
**Haunstetten, Postillionstraße**	**30**	**Massy**	**111**	**EBA**	**Rudernadel w/ large head**	**1.2**	**16.4**	**43.8**	**3.1**	**-21.1**	**9.6**
**Haunstetten, Postillionstraße**	**41**	**Massy**	**2**	**EBA**	**Rudernadel w/ large head**	**2.1**	**17.1**	**43.0**	**2.9**	**-20.7**	**9.0**
**Königsbrunn, Obere Kreuzstraße**	**14**	**Massy**	**5**	**EBA**	**Rudernadel w/ large head**	**2.7**	**14.3**	**37.5**	**3.1**	**-20.2**	**10.0**
**Singen, Nordstadtterrasse, Lessingstrasse**	**7**	**Krause**	**55/24**	**EBA**	**Rudernadel w/ large head**	**2.2**	**12.4**	**40.3**	**3.8**	**-20.9**	**9.7**
**Singen, Nordstadtterrasse, Widerholtstrasse**	**65**	**Krause**	**53/4**	**EBA**	**Rudernadel w/ large head**	**5.5**	**14.6**	**42.8**	**3.4**	**-20.6**	**9.9**
**Haunstetten, Postillionstraße**	**8**	**Massy**	**140**	**EBA**	**Bone pin/boar tusk pin**	**1.5**	**14.6**	**39.0**	**3.1**	**-20.5**	**9.1**
**Haunstetten, Postillionstraße**	**16**	**Massy**	**84**	**EBA**	**Bone pin/boar tusk pin**	**2.6**	**15.9**	**42.6**	**3.1**	**-21.0**	**10.1**
**Haunstetten, Postillionstraße**	**36**	**Massy**	**6**	**EBA**	**Bone pin/boar tusk pin**	**0.8**	**15.9**	**40.1**	**2.9**	**-20.7**	**9.1**
**Haunstetten, Unterer Talweg 58–62**	**4**	**Massy**	**152**	**EBA**	**Bone pin/boar tusk pin**	**2.4**	**16.6**	**45.8**	**3.2**	**-21.3**	**8.8**
**Königsbrunn, Obere Kreuzstraße**	**17**	**Massy**	**50**	**EBA**	**Bone pin/boar tusk pin**	**1.4**	**16.6**	**45.0**	**3.2**	**-20.4**	**9.3**
**Königsbrunn, Obere Kreuzstraße**	**25**	**Massy**	**67**	**EBA**	**Bone pin/boar tusk pin**	**2.2**	**16.7**	**45.3**	**3.1**	**-19.8**	**10.6**
**Königsbrunn, Obere Kreuzstraße**	**29**	**Massy**	**93**	**EBA**	**Bone pin/boar tusk pin**	**5.7**	**17.0**	**45.3**	**3.1**	**-20.6**	**9.8**
**Königsbrunn, Obere Kreuzstraße**	**39**	**Massy**	**82**	**EBA**	**Bone pin/boar tusk pin**	**2.1**	**16.8**	**45.0**	**3.2**	**-20.0**	**10.6**
**Königsbrunn, Obere Kreuzstraße**	**43**	**Massy**	**86**	**EBA**	**Scheibenkopfnadel**	**2.3**	**15.1**	**41.0**	**3.2**	**-20.3**	**9.7**
**Königsbrunn, Obere Kreuzstraße**	**46**	**Massy**	**85**	**EBA**	**Scheibenkopfnadel**	**5.6**	**16.2**	**46.1**	**3.1**	**-20.4**	**10.4**
**Wehringen, Hochfeld**	**6**	**Massy**	**1380**	**EBA**	**Schleifenkopfnadel**	**1.6**	**11.7**	**32.2**	**3.2**	**-20.78**	**9.98**
**Wehringen, Hochfeld**	**8**	**Massy**	**1586**	**EBA**	**Schleifenkopfnadel**	**4.1**	**16.4**	**44.9**	**3.2**	**-20.16**	**10.66**
**Wehringen, Hochfeld**	**3**	**Massy**	**1564**	**EBA**	**Horkheimer Nadel**	**6.5**	**17.2**	**45.7**	**3.1**	**-20.78**	**10.49**
**Wehringen, Hochfeld**	**7**	**Massy**	**1474**	**EBA**	**Horkheimer Nadel**	**5.5**	**17.1**	**46.8**	**3.2**	**-20.95**	**10.65**

To conclude: at least for the Augsburg region, our data propose an absolute date for Bz A from 2150/2100 BC until at least 1700 BC. Bz A1a and Bz A2a pin types indeed represent a sequence (Fig 7). It seems that the so-called Bz A1b pin types are used during the complete EBA and cannot be used for any chronological subdivision. Compared with the traditional dating of Bz A between 2300/2200 BC and 1600/1550 BC, our results imply a substantial narrowing of the duration of the Early Bronze Age from 750/700 years to possibly 450 years. The MBA, however, seems to start already in the 17^th^ century. Therefore, even if we have not been able to date graves with Bz A2b or Bz A2c type objects, there is not much time for a continuation of Bz A into the 17^th^ century, unless one supposes a period of overlap between Bz A and Bz B.

### The cemetery of Singen

**Fig 9:**
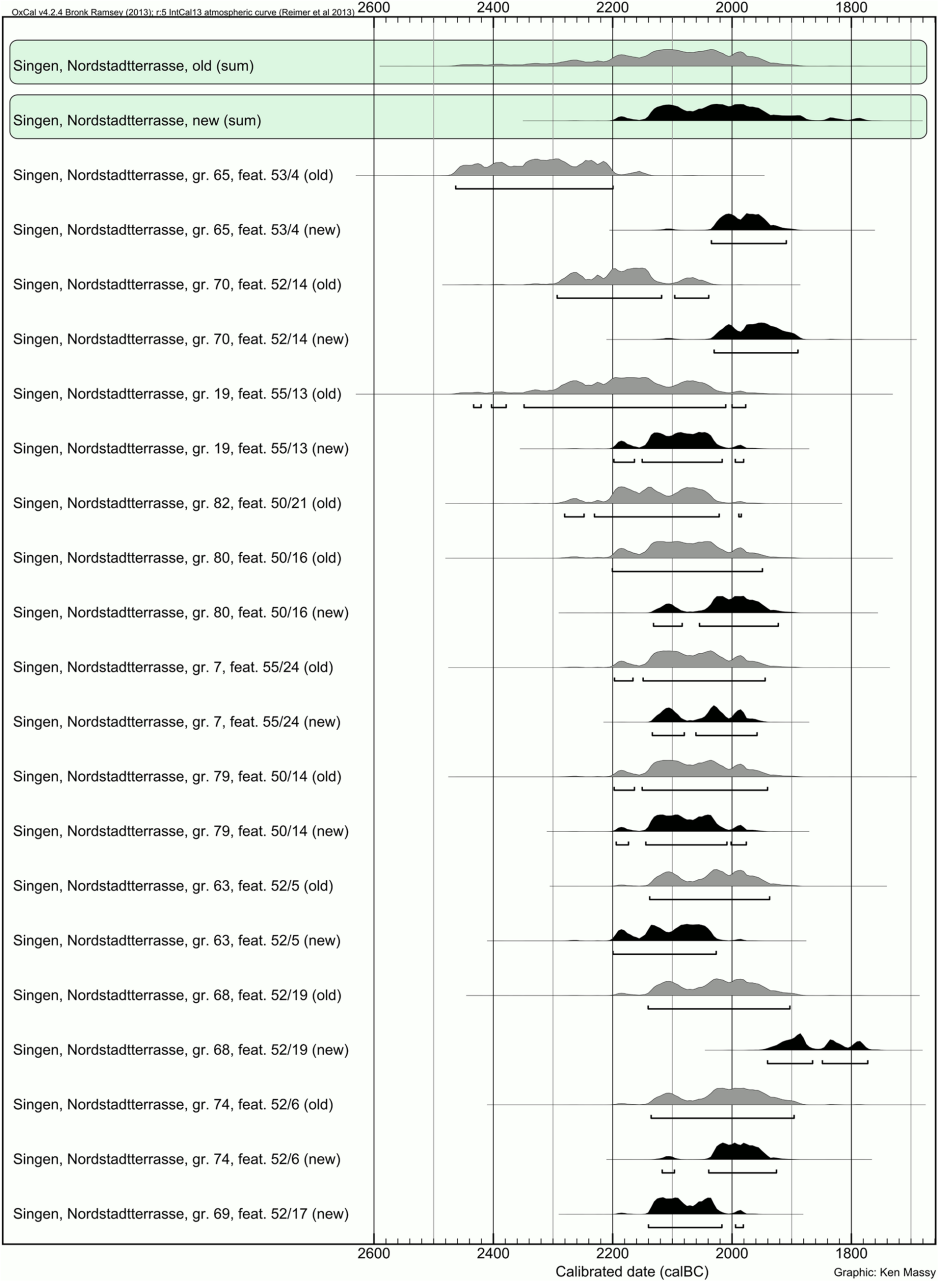
Plot of old (grey) and new (black) radiocarbon dates from the cemetery of Singen. The sum calibration is shown on top.

Our (re-)evaluation of 10 graves from Singen revealed surprising results (Fig 9). The original dating of five graves (80, 7, 79, 74, 63) (Table 4) was confirmed in our study. The re-dating of grave 68 has to be considered with caution. Although the C:N ratio points to a good quality of the extracted collagen, the overall amount of carbon in the sample (4.7%) is rather low and there still is a chance that not all conservation chemicals have been removed. Most interestingly, the three earliest dates of the original series, i.e. graves 65, 70 and 19, shifted to a much younger date. The reason for this discrepancy remains unclear. It is possible that curators treated some of the bones immediately after the excavation and before the first sampling for ^14^C analysis. It should be noted that for radiometric ^14^C dating in the 1980ies rather large quantities of bone (50 grams and more) were required, which severely limits selective sampling to avoid contaminated bone. In contrast, the small sample size for AMS dating (typically 1 gram) allows for more rigorous sampling. In addition the ultrafiltration step in the AMS pretreatment sequence may have eliminated contamination more reliably. The observation that 5 out of 8 results agree well between the two techniques may in fact indicate the potential of contamination for the three older radiometric dates.

**Table 4:** Burials from Singen with old and new radiocarbon dates (na = not analyzed).

Name of site	GraveNo.	GraveNo. after	Feat.No.	LaborNo.	C14 Age	±	Cal 1 sigma	Cal 2 sigma	C:N	%C	LaborNo. (from year 1988)	C14 Age (from year 1988)	± (from year 1988)
Singen, Nordstadtterrasse, Goethestrasse	63	Krause	52/5	MAMS 21968	3712	29	cal BC 2188–2039	cal BC 2199–2028	3.1	36.6	HD—10692	3655	35
Singen, Nordstadtterrasse, Lessingstrasse	7	Krause	55/24	MAMS 21974	3662	21	cal BC 2123–1980	cal BC 2133–1959	3.1	6.6	HD 8972–9116	3679	42
Singen, Nordstadtterrasse, Lessingstrasse	19	Krause	55/13	MAMS 21967	3700	30	cal BC 2136–2036	cal BC 2197–1981	3.0	30.0	HD 8973–9117	3756	60
Singen, Nordstadtterrasse, Widerholtstrasse	65	Krause	53/4	MAMS 21975	3620	22	cal BC 2022–1947	cal BC 2035–1910	na	na	HD 8974–9155	3847	43
Singen, Nordstadtterrasse, Widerholtstrasse	68	Krause	52/19	MAMS 21973	3537	22	cal BC 1920–1783	cal BC 1941–1774	3.3	4.7	HD 8975–9145	3651	43
Singen, Nordstadtterrasse, Widerholtstrasse	69	Krause	52/17	MAMS 21976	3686	21	cal BC 2131–2033	cal BC 2139–1982	3.3	24.0	na	na	na
Singen, Nordstadtterrasse, Widerholtstrasse	70	Krause	52/14	MAMS 21969	3604	29	cal BC 2016–1922	cal BC 2029–1892	2.9	25.3	HD 8978–9157	3766	37
Singen, Nordstadtterrasse, Widerholtstrasse	74	Krause	52/6	MAMS 21972	3632	21	cal BC 2025–1963	cal BC 2116–1926	3.1	17.7	HD 8976–9129	3638	44
Singen, Nordstadtterrasse, Widerholtstrasse	79	Krause	50/14	MAMS 21970	3688	29	cal BC 2134–2032	cal BC 2194–1977	3.0	25.4	HD 8971–9115	3677	44
Singen, Nordstadtterrasse, Widerholtstrasse	80	Krause	50/16	MAMS 21971	3641	29	cal BC 2106–1952	cal BC 2132–1922	3.0	31.8	HD 8970–9147	3690	46
Singen, Nordstadtterrasse, Widerholtstrasse	82	Krause	50/21	na	na	na	na	na	na	na	HD—10691	3730	40

Our results force us to lower the starting point of the Singen cemetery from 2300 BC to 2200 BC at the earliest (with grave 63 as the oldest: 2200–2027 BC with 95.4% probability). It is even more likely that Singen rather started around 2150 BC (see sum calibration Fig 9). Excluding the unreliable result for grave 68, the latest 2σ calibrated ranges end around 1900 BC.

### Comparison of Augsburg region, Singen and the Neckar region

Our new results from Singen fit very well to the results from the Augsburg region. The oldest dates from Singen (grave 63: 3712±12 ^14^C BP) and the Augsburg region (Haunstetten, Postillonstraße, grave 5: 3717±23 ^14^C BP) are almost identical as is the period of use of Singen and Haunstetten, Postillonstraße.

The dates for Bz A type objects from both regions provide a coherent view (Fig 6). The *Ruderkopfnadeln* (with small or large head) are identical in date and seem to have been used in both places at the same time. The much later date for Singen, grave 65, and the *Ruderkopfnadel* with large head from the grave fit perfectly to the time span for this type of pin from the Augsburg region. A difference can be seen in the early date for a *Horkheimer Nadel* from Singen, grave 79, which suggests a use of this type object in the 21^st^ and possibly already in the late 22^nd^ century BC, and, therefore, clearly earlier than in the Augsburg region. As the old dating of grave 79 was confirmed by the new measurement, the early position of this *Horkheimer Nadel* is beyond any doubt. Taking together the dating of the *Horkheimer Nadel*, shows a surprisingly long use of this particular type of pin, which spans a large part of the EBA.

Due to the lack of Bz A2 type objects at Singen, the cemetery does not allow further insights into the chronological sequence beyond Bz A1. However, Singen reconfirms the parallel existence of Bz A1a and Bz A1b.

**Fig 10:**
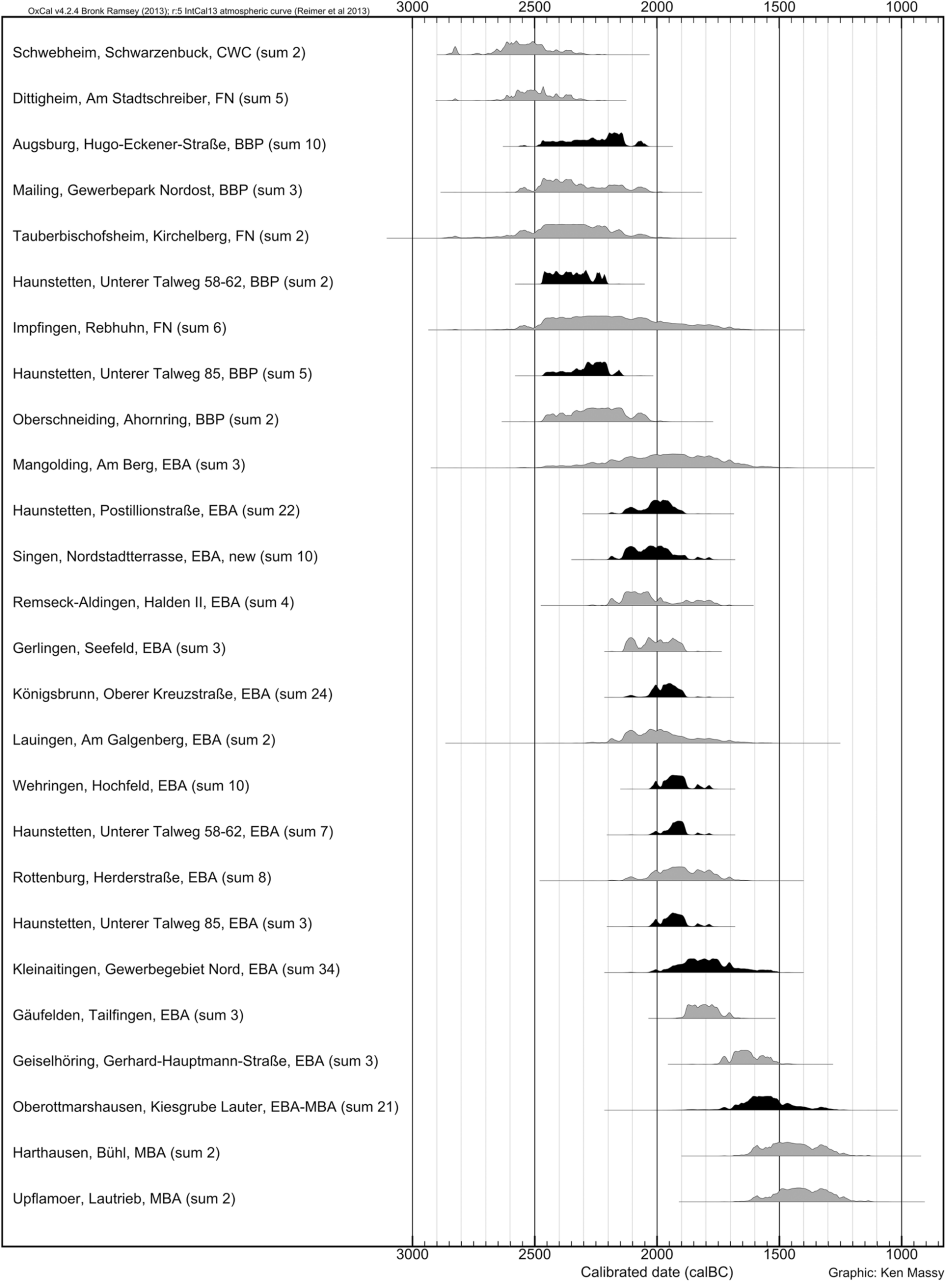
Sum calibrations of cemeteries from south Germany. Dates made in our project are marked in black.

The radiocarbon dates for the Neckar region around the city of Stuttgart (Fig 1) also match our results from Augsburg and Singen (Fig 10) [20]. Our new data show that the dates from the Neckar region do not contradict the chronological development as has long been assumed but fit very well into the overall picture.

There are only three graves with Bz A pins which were dated by the radiometric technique. In the Neckar region, two ^14^C dated graves contain Bz A1 type objects, i.e. a *Scheibenkopfnadel* or *Ruderkopfnadel* (Gäufelden, grave 1, individual 2; Remseck-Aldingen, Halden II, gr. 15); however, in both cases the state of preservation does not allow a further taxonomic identification of the pins which can, therefore, be attributed either to Bz A1a or Bz A1b following the Ruckdeschel system. The related 2σ calibrated ranges (Gäufelden, grave 1, individual 2: 1882–1747 BC with 95.4% probability; Remseck-Aldingen, Halden II, gr. 15: 1936–1746 BC with 95.4% probability) fit into the long time span for Bz A1 type objects between 2150 BC and 1700 BC.

The third burial, Rottenburg, Herderstraße, grave 1, contained a singular type of pin which does not appear in the Ruckdeschel system, but which Rüdiger Krause proposes to relate to Bz A2 shapes [25]. Due to the significant standard deviation, this old date (2137–1768 BC with 95.4% probability) does not contribute to a better understanding of the EBA chronology as it spans almost the complete EBA.

To conclude: the few radiocarbon dated burials with Bz A type objects are consistent with the late Bz A1 dates from Augsburg.

## Discussion

Given our results from Augsburg, we can contribute to the long-standing discussion on the transition from the LN to the EBA in Central Europe. For eastern Germany, Johannes Müller [55] postulated a co-existence of LN and EBA groups for at least 150 years. Regarding southern Germany, Volker Heyd [56] already postulated a transition from the LN to EBA without any overlap around 2150/2100 BC, however, on the basis of only a small number of radiocarbon dates from Late Neolithic burials. Our data confirm that there is neither a significant gap nor long overlap between both periods. The radiocarbon dates from the cemeteries of Augsburg, Hugo-Eckener-Straße, and Haunstetten, Postillonstraße clearly show a succession from latest LN to earliest EBA burials shortly after 2150 BC. This fits very well to recent radiocarbon dates for the Bell Beaker settlement of Engen-Welschingen, Guuhaslen, and its relation to our new dates for nearby Singen: the 2σ calibrated ranges of the three dates from Welschingen fall between 2490 BC and 2110 BC [57] and the EBA at Singen probably does not start before 2150 BC.

In summary: a very short overlap or gap between the LN and the EBA in southern Germany is possible, but a smooth transition seems more plausible.

Our results fundamentally question the absolute and relative chronology of the EBA and its inherent notion of a linear and gradual development for southern Germany. Since Paul Reinecke [3] the subdivision of the EBA into the phases Bz A1 and Bz A2 was explained with the growing ability to manage the new bronze technology. Almost all further chronological discussion has kept the view of a gradual development of the technology from bone and boar tusk pins to the first hammered metal objects–consisting mostly of copper with hardly any tin–in Bz A1 up to perfectly alloyed bronze with 90% of copper and 10% of tin and refined casting techniques in Bz A2 [14], [15]. With this in mind, a sequence of pin types was proposed which again was taken as a basis for socio- and cultural-historical interpretation. It was taken for granted that the duration of use and deposition of each type was rather restricted to a short period of time. Simultaneously, it was assumed that the similarity of particular types of pins also indicates contemporaneity.

The results from the Augsburg region and Singen both refute a any linear and gradual development and rather indicate very different dynamics in the late 3^rd^ and early 2^nd^ millennium. The sequence of pins is far more complex than proposed. Bone and boar tusk pins are not amongst the earliest EBA pin types and probably continue into the early 19^th^ century BC in the Augsburg region. Pin types defined as Bz A1a by Ruckdeschel [15] indeed start with the beginning of the EBA–but so do also the pins of his sub-phase Bz A1b, which he supposed to succeed Bz A1a. Whereas the Bz A1a pin types indeed end around 1900 BC, Bz A1b pin types continue until the end of the EBA around 1700 BC (Fig 6), (Fig 7). In contrast to the dominant notion of research, Bz A2 pins do not replace the simple hammered pins of Bz A1. This is most obvious in Kleinaitingen, where Bz A1b and Bz A2a pins were deposited in contemporaneous burials. Bz A2a type objects start around 1900 BC and are used and deposited parallel to Bz A1b pin types until 1700 BC. However, it has to be kept in mind that we were not able to date type objects of Bz A2b and Bz A2c, which might indicate a continuation of the EBA after 1700 BC. Therefore, the traditional notion of the sequence of Bz A1 and Bz A2 and the associated idea of the development from simple hammered pins (“*Blechkreis*”) to complex cast pins have to be revised.

Our data also shed new light on the transition from the EBA to the MBA. Up to now, a start of the MBA in Southern Germany around 1550 BC has been taken for granted (i.a. [21], [24]). So far, we have only five radiocarbon dated graves with MBA type objects from the Augsburg region, two of which can securely be attributed to the earliest MBA (Bz B) on the basis of particular types of pins. It seems that the MBA at least in the Augsburg region started significantly earlier than it has been proposed so far as the 2σ calibrated range of the Bz B inventory from Oberottmarshausen, grave 5, mostly falls into the 17^th^ century BC. If such an early start of the MBA could be demonstrated on a larger basis of analyses, this would have a major impact on historical narratives in Central Europe. Such a re-dating would solve the problem that at this point the Central European MBA seems to be a rather short period regarding the fundamental social changes which took place. Moreover, the deposition of the Nebra hoard (1639–1401 BC with 95.4% probability) [58] and the related dating of the Apa horizon (with two swords of Apa type in the Nebra hoard) would then be contemporaneous with the MBA. However, only more radiocarbon dated Bz B graves will allow us to better determine the chronological position of this phase.

The contemporaneity of Bz A1 and Bz A2a as well as the small number of Bz A2 pins in the Augsburg region and in southern Germany as a whole raise the question, how to explain the appearance of these bronzes in this larger region. It seems that Bz A2a objects, most of which are related to the Únětice culture, should be interpreted as the appropriation of foreign influences and objects in southern Germany, which basically “stayed Bz A1” during the complete EBA. The Bz A2a Únětice bronzes could rather be seen as supplement to the local inventory of the material culture.

Moreover, it is most likely that Bz A2 in the area of the Únětice culture started considerably earlier than in the Augsburg region. An early start for Bz A2a already in the late 3^rd^ millennium or at least around 2000 BC is indicated by Quenstedt, grave 34, a grave from Feuersbrunn and possibly also hoard II of Melz (cf. also the princely grave of Leubingen with a dendrodate of 1942±10 BC [23]). However, the rarity of metal objects as grave goods in the Únětice region poses a serious problem to a better understanding of the chronological development of the Únětice culture. Bz A1 bronzes are extremely rare in this area. Bz A2a type objects continue at least until the late 18^th^ century BC (cf. [59]: Prag-Miškovice, grave 32: *Ösenkopfnadel*, 1950–1740 BC with 95.4% probability), where they sometimes appear contemporaneously with Bz A1 pin types in the same cemetery (cf. [59]: Prag-Miškovice, grave 18: *Schleifenkopfnadel*, 1970–1740 BC with 95.4% probability). This could indicate a very similar situation to what we are able to document in the Augsburg region.

## Conclusions

For almost a hundred years, Early Bronze Age chronology has been dominated by an evolutionist paradigm that assumed a linear development from simple to elaborate bronze objects, whose chronological placement was based on a small number of radiocarbon dated burials since the 1980s. With a large corpus of new radiocarbon dated Late Neolithic, Early and Middle Bronze Age graves from the Augsburg region, we were able to shed new light on the cultural dynamics in the late 3^rd^ and first half of the 2^nd^ millennium BC.

We have demonstrated that the transition from the LN to the EBA happened without a significant gap or overlap in southern Germany around 2150 BC. Our results suggest that the time span of the EBA has to be shortened from conventionally postulated around 750/700 years to only about 450 years–from 2150 BC to at least 1700 BC. During the complete span of time, so-called Bz A1 pin types were used and deposited. More complex objects dated to Bz A2 appear around 1900 BC for the first time. Therefore, we do not see any transition from Bz A1 to Bz A2 in the way it has been proposed for the last 100 years. Our data show a complex coexistence of different types as well as simple and sophisticated shapes of bronze objects. We propose that Bz A1 and Bz A2 should not be understood as a chronological sequence even beyond southern Germany. In our view, Bz A1 and Bz A2 are nothing more than the consequence of different rates of appropriation of bronze technologies in southern Germany (= Bz A1) and the region of the Únětice culture in eastern Germany, Bohemia, Moravia, western Poland and parts of Slovakia and Austria (= Bz A2).

In summary, Bz A1 and Bz A2 seem to appear as different levels of ability and willingness to appropriate the new bronze technology and the non-technological knowledge transferred together with the technological knowledge. Bz A1 and Bz A2 do not represent chronological but rather regional phenomena. Bz A2 finds in southern Germany and Bz A1 finds in the area of the Únětice culture should be explained as the local appropriation of foreign objects rather than autonomous chronological phases.

## References

[1] Childe VG. The dawn of European civilization. London: K. Paul; 1925.

[2] Reinecke P. Beiträge zur Kenntnis der frühen Bronzezeit Mitteleuropas. Mitteilungen der Anthropologischen Gesellschaft Wien. 1902; 32: 104–154.

[3] Reinecke P. Zur chronologischen Gliederung der süddeutschen Bronzezeit. Germania. 1924; 8: 43–44.

[4] Pare ChFE. Bronze and the Bronze Age In: Pare ChFE, editor. Metals Make the World Go Round The Supply and Circulation of Metals in Bronze Age Europe. Oxford: Oxbow; 2000: 1–38.

[5] Kienlin TL. Frühes Metall im nordalpinen Raum: Eine Untersuchung zu technologischen und kognitiven Aspekten früher Metallurgie anhand der Gefüge frühbronzezeitlicher Beile. Univärsitätsforschungen zur Prähistorischen Archäologie 162. Bonn: Habelt-Verlag; 2008.

[6] Kienlin TL. Traditions and Transformations Approaches to Eneolithic (Copper Age) and Bronze Age Metalworking and Society in Eastern Central Europe and the Carpathian Basin. BAR Internat. Ser. 2184 Oxford: Archaeopress; 2010.

[7] Ch Strahm. Prestige versus Ingenium. Die Beweggründe für die Entwicklung der Metallurgie In: Grunwald S/Koch JK/Mölders D/Sommer U/Wolfram S, editors. Artefact. Festschrift S. Rieckhoff. Univärsitätsforschungen zur Prähistorischen Archäologie 172. Bonn: Habelt-Verlag; 2009: 343–353.

[8] Mandera H-E. Versuch einer Gliederung der Aunjetitzer Kultur in Mitteldeutschland. Jahresschr. Halle. 1953; 37: 177–236.

[9] Pleinerova I. Únětická kultura v oblasti Krušných hor a jejich sousedství II, Památky archeologické. 1967; 58: 1–36.

[10] Müller J. Zur absolutchronologischen Datierung und Interpretation der mitteldeutschen Aunjetitz-Inventare In: Bátora J, Peška J, editors. Aktuelle Probleme der Erforschung der Frühbronzezeit in Böhmen und Mähren und in der Slowakei. Nitra: Archäologisches Institut der Slowakischen Akademie der Wissenschaften; 1999: 113–126.

[11] Bartelheim M. Studien zur böhmischen Aunjetitzer Kultur Chronologische und chorologische Untersuchungen. Univärsitätsforschungen zur Prähistorischen Archäologie 46. Bonn: Habelt-Verlag; 1998.

[12] von Brunn WA. Bronzezeitliche Hortfunde I Die Hortfunde der frühen Bronzezeit aus Sachsen-Anhalt, Sachsen und Thüringen. Deutsche Akademie der Wissenschaften zu Berlin, Schriften der Sektion für Vor- und Frühgeschichte 7. Berlin: Akademie-Verl; 1959.

[13] Zich B. Studien zur regionalen und chronologischen Gliederung der nördlichen Aunjetitzer Kultur Vorgeschichtliche Forschungen 20. Berlin/New York: De Gruyter; 1996.

[14] Christlein R. Beiträge zur Stufengliederung der frühbronzezeitlichen Flachgräber in Süddeutschland. Bayerische Vorgeschichtsblätter. 1964; 27: 25–63.

[15] Ruckdeschel W. Die frühbronzezeitlichen Gräber Südbayerns Ein Beitrag zur Kenntnis der Straubinger Kultur. Antiquitas II Bonn: Habelt-Verlag; 1978.

[16] Möslein S. Die Straubinger Gruppe der donauländischen Frühbronzezeit: Frühbronzezeitliche Keramik aus Südostbayern und ihre Bedeutung für die chronologische und regionale Gliederung der frühen Bronzezeit in Südbayern. Berichte der Bayerischen Bodendenkmalpflege. 1998; 38: 37–106.

[17] David W. Zu früh- und ältermittelbronzezeitlichen Grabfunden in Ostbayern In: Michálek J, Schmotz K, Zápotocká M, editors. 7. Treffen, Archäologische Arbeitsgemeinschaft Ostbayern/West- und Südböhmen, Landau an der Isar, Juni 1997. Rahden/Westf: Verlag Marie Leidorf; 1998: 108–129.

[18] Gerloff S. Von Troja an die Saale, von Wessex nach Mykene. Chronologie, Fernverbindungen und Zinnrouten der Frühbronzezeit Mittel- und Westeuropa. In: Meller H, Bertemes F, editors. Der Griff nach den Sternen. Wie Europas Eliten zu Macht und Reichtum kamen. Internationales Symposium Halle, 16.–21. Februar 2005. Halle: Tagungen des Landesmuseums für Vorgeschichte Halle 5–2; 2010: 603–639.

[19] Krause R. Die endneolithischen und frühbronzezeitlichen Grabfunde auf der Nordstadtterrasse von Singen am Hohentwiel Forschungen und Berichte zur Vor- u Frühgeschichte in Baden-Württemberg 32. Stuttgart: Konrad Theiss Verlag; 1988.

[20] Becker B, Krause R, Kromer B. Zur absoluten Chronologie der Frühen Bronzezeit. Germania. 1989; 67: 421–442.

[21] Hafner A, Suter PJ. Vom Endneolithikum zur Frühbronzezeit. Wandeln und Kontinuität zwischen 2400 und 1500 v. Chr. Archäologisches Korrespondenzblatt. 2003; 33: 325–344.

[22] Krause R. Studien zur kupfer- und frühbronzezeitlichen Metallurgie zwischen Karpatenbecken und Ostsee. Rahden/Westf: Verlag Marie Leidorf; 2003.

[23] Becker B, Jäger KD, Kaufmann D, Litt T. Dendrochronologische Datierungen von Eichenhölzern aus den frühbronzezeitlichen Hügelgräbern bei Helmsdorf und Leubingen (Aunjetitzer Kultur) und an bronzezeitlichen Flußeichen bei Merseburg. Jahresschrift für mitteldeutsche Vorgeschichte. Halle 1989; 72: 299–312.

[24] Müller J, Lohrke B. Neue absolutchronologische Daten für die süddeutsche Hügelgräberbronzezeit. Germania. 2009; 87–1: 25–39.

[25] Krause R. Zur Chronologie der frühen und mittleren Bronzezeit Süddeutschlands, der Schweiz und Österreichs. In: Randsborg K, editor. Absolute chronology: archaeological Europe 2500–500 B. C. Acta Archaeologica 1996; 67: 73–86.

[26] Rassmann K. Zum Forschungsstand der absoluten Chronologie der frühen Bronzezeit in Mitteleuropa auf der Grundlage von Radiocarbondaten. In: Randsborg K, editor. Absolute chronology: archaeological Europe 2500–500 B. C. Acta Archaeologica 1996; 67: 199–209.

[27] Lorenz L. Typologisch-chronologische Studien zu Deponierungen der nordwestlichen Aunjetitzer Kultur Univärsitätsforschungen zur Prähistorischen Archäologie 188. Bonn: Habelt-Verlag; 2010.

[28] Schwenzer S. Zur Frage der Datierung der Melzer Stabdolche. Prähistorische Zeitschrift. 2002; 77: 76–83.

[29] Gerloff S. Zu Fragen mittelmeerländischer Kontakte und absoluter Chronologie der Frühbronzezeit in Mittel- und Westeuropa. Prähistorische Zeitschrift. 1993; 68: 58–102.

[30] Strahm C. Das Kulturenkonzept und das Periodisierungskonzept. Ein methodischer Beitrag zur Gliederung und Dynamik der Frühbronzezeit In: Eberschweiler B, Köninger J, Schlichtherle H, Strahm C. Aktuelles zur Frühbronzezeit und frühen Mittelbronzezeit nördlich der Alpen. Rundgespräch Hemmenhofen 6. Mai 2000. Hemmenhofener Skripte 2. Freiburg im Breisgau: Janus-Verlag; 2001: 177–184.

[31] Kromer B. 14C-Datierung der Knochenproben aus Singen am Hohentwiel In: Krause R., Die endneolithischen und frühbronzezeitlichen Grabfunde auf der Nordstadtterrasse von Singen am Hohentwiel. In: Krause R. Die endneolithischen und frühbronzezeitlichen Grabfunde auf der Nordstadtterrasse von Singen am Hohentwiel. Forschungen und Berichte zur Vor- und Frühgeschichte in Baden-Württemberg 32 Stuttgart: Konrad Theiss Verlag; 1988: 250–251.

[32] Kromer B, Münnich KO. CO2 gas proportional counting in radiocarbon dating—review and perspective In: Taylor RE, Long A, Kra RS. Radiocarbon after Four Decades. New York: Springer; 1992: 184–197.

[33] Longin R. New method of collagen extraction for radiocarbon dating. Nature 1971; 230, 241–242. 4926713 10.1038/230241a0

[34] Kromer B, Lindauer S, Synal HA, Wacker L. MAMS–A new AMS facility at the Curt-Engelhorn-Centre for Achaeometry, Mannheim, Germany. Nucl Instrum Methods Phys Res B. 2013; 294: 11–13.

[35] Ascough PL, Cook GT, Dugmore AJ, Barber J, Higney E, Scott EM. Holocene variations in the Scottish marine radiocarbon reservoir effect. Radiocarbon. 2004; 46–2: 611–620.

[36] Bronk Ramsey C. Bayesian analysis of radiocarbon dates. Radiocarbon. 2009; 51–1: 337–360.

[37] Reimer PJ, Bard E, Bayliss A, Beck JW, Blackwell PG, Ramsey CB, et al IntCal13 and Marine13 Radiocarbon Age Calibration Curves 0–50,000 Years cal BP. Radiocarbon. 2013; 55–4: 1869–1887.

[38] Buck CE, Kenworthy JB, Litton CD, Smith AFM. Combining archaeological and radiocarbon information: a Bayesian approach to calibration. Antiquity. 1991; 65–249: 808–821.

[39] Geyh MA. An overview of 14C analysis in the study of groundwater. Radiocarbon. 2000; 42–1: 99–114.

[40] Ascough PL, Church MJ, Cook GT, Dunbar E, Gestsdóttir H, et al Radiocarbon reservoir effects in human bone collagen from northern Iceland. Journal of Archaeological Science. 2012; 39–7: 2261–2271.

[41] Milner N, Craig OE, Bailey GN, Pedersen K, Andersen SH. Something fishy in the Neolithic? A re-evaluation of stable isotope analysis of Mesolithic and Neolithic coastal populations. Antiquity. 2004; 78: 9–22.

[42] Koch JK, Kupke K. Life-course reconstruction for mobile individuals in an Early Bronze Age society in Central Europe: Concept of the project and first results for the cemetery of Singen (Germany) In: Kaiser E, Burger J, Schier W, editors. Population dynamics in prehistory and early history New approaches using stable isotopes and genetics. Berlin: deGruyter; 2012: 225–240.

[43] Kupke K. Ernährungsrekonstruktion mittels Kohlenstoff- und Stickstoffisotopen aus dem frühbronzezeitlichen Gräberfeld von Singen, Kr. Konstanz und den früheisenzeitlichen Gräbern im Magdalenenberg bei Villingen, Schwarzwald-Baar-Kreis. Magisterarbeit zur Erlangung des akademischen Grades Magister Artium (M.A.). Universität Leipzig. 2010.

[44] Paul D, Skrzypek G, Fórizs I. Normalization of measured stable isotopic compositions to isotope reference scales–a review. Rapid Communications in Mass Spectrometery. 2007; 21–18: 3006–3014. 10.1002/rcm.3185 17705258

[45] Innerhofer F. Die mittelbronzezeitlichen Nadeln zwischen Vogesen und Karpaten Studien zur Chronologie, Typologie und regionalen Gliederung der Hügelgräberkultur. Univärsitätsforschungen zur Prähistorischen Archäologie 71 Bonn: Habelt-Verlag; 2000.

[46] David-Elbiali M. La Suisse occidentale au IIe millénaire av. J.-C.: chronologie, culture et intégration européenne Cahiers d’archéologie romande 80. Lausanne: Bibliothèque historique vaudoise; 2000.

[47] Vogt I. Der Übergang von der frühen zur mittleren Bronzezeit in Mittel- und Nordeuropa unter besonderer Berücksichtigung der Griffplattenklingen. Bonn: Habelt-Verlag; 2004.

[48] Pospieszny Ł. Freshwater reservoir effect and the radiocarbon chronology of the cemetery in Ząbie, Poland. Journal of Archaeological Science. 2015; 53: 264–276.

[49] Shishlina NI, Zazovskaya EP, van der Plicht J, Sevastyanov VS. Isotopes, plants, and reservoir effects: Case studies from the Caspian Bronze Age. Radiocarbon. 2012; 54–3,4: 749–760.

[50] Asam T, Grupe G, Peters J. Human subsistence strategy in Neolithic times: an isotopic analysis of Bavarian skeletal findings. Anthropologischer Anzeiger. 2006; 64–1: 1–23. 16623085

[51] Asam T, Bösl C, Grupe G, Lösch S, Manhart H, Mekota AM, et al Palaeoecosystem reconstruction and the Neolithic Transition in temperate climates In: Grupe G, Peters J, editors. Documenta Archaeobiologiae 2. Conservation policy and current research–Grundsätze der Bewahrung und aktuelle Forschung. Rahden/Westf.: Verlag Marie Leidorf; 2005: 97–137.

[52] Fuller BT, Müldner G, van Neer W, Ervynck A, Richards MP. Carbon and nitrogen stable isotope ratio analysis of freshwater, brackish and marine fish from Belgian archaeological sites (1st and 2nd millennium AD). Journal of Analytical Atomic Spectrometry. 2012; 27: 807–820.

[53] Bogaard A, Heaton THE, Poulton P, Merbach I. The impact of manuring on nitrogen isotope ratios in cereals: archaeological implications for reconstruction of diet and crop management practices. Journal of Archaeological Science. 2007; 34–3: 335–343.

[54] Kanstrup M, Thomsen IK, Andersen AJ, Bogaard A, Christensen BT. Abundance of 13C and 15N in emmer, spelt and naked barley grown on differently manured soils: towards a method for identifying past manuring practice. Rapid Communications in Mass Spectrometery. 2011; 25–19: 2879–2887. 10.1002/rcm.5176 21913266

[55] Müller J. Radiokarbonchronologie, Keramiktechnologie, Osteologie, Anthropologie, Raumanalysen. Beiträge zum Neolithikum und zur Frühbronzezeit im Mittelelbe-Saale-Gebiet. Bericht der Römisch-Germanischen Kommission. 2001; 80: 25–211.

[56] Heyd V. Die Spätkupferzeit in Süddeutschland Untersuchungen zur Chronologie von der ausgehenden Mittelkupferzeit bis zum Beginn der Frühbronzezeit im süddeutschen Donaueinzugsgebiet und den benachbarten Regionen bei besonderer Berücksichtigung der keramischen Funde. Bonn: Habelt-Verlag; 2000.

[57] Lechterbeck J, Kerig T, Kleinmann A, Sillmann M, Wick L, Rösch. How was Bell Beaker economy related to Corded Ware and Early Bronze Age lifestyles? Archaeological, botanical and palynological evidence from the Hegau, Western Lake Constance region. Environmental Archaeology. 2014; 19–2: 95–113.

[58] Meller H. Die neolithischen und bronzezeitlichen Goldfunde Mitteldeutschlands—Eine Übersicht. In: Meller H/Risch R/Pernicka E, editors. Metalle der Macht—Frühes Gold und Silber. 6. Mitteldeutscher Archäologentag vom 17. bis 19. Oktober 2013 in Halle (Saale). Halle: Tagungen des Landesmuseums für Vorgeschichte Halle 11–2; 2014: 696.

[59] Ernée M, Müller J, Rassmann K. Ausgrabung des frühbronzezeitlichen Gräberfelds der Aunjetitzer Kultur von Prag-Miškovice. Vorläufige Auswertung und erste Ergebnisse der naturwissenschaftlichen Untersuchungen, 14C-Daten und Metallanalysen. Germania. 2009; 87: 355–410.

